# Molecular mechanisms of *Shigella* effector proteins: a common pathogen among diarrheic pediatric population

**DOI:** 10.1186/s40348-022-00145-z

**Published:** 2022-06-19

**Authors:** Ahmad Nasser, Mehrdad Mosadegh, Taher Azimi, Aref Shariati

**Affiliations:** 1 Department of Pathobiology, School of Public Health, Tehran University of Medical Sciences, Tehran, Iran; 2 Department of Bacteriology & Virology, School of Medicine, Shiraz University of Medical Sciences, Shiraz, Iran; 3 Molecular and medicine research center, Khomein University of Medical Sciences, Khomein, Iran

**Keywords:** *Shigella*, Toxin, Effector proteins, Immune response, Pathogenesis, Children

## Abstract

Different gastrointestinal pathogens cause diarrhea which is a very common problem in children aged under 5 years. Among bacterial pathogens, *Shigella* is one of the main causes of diarrhea among children*,* and it accounts for approximately 11% of all deaths among children aged under 5 years. The case-fatality rates for *Shigella* among the infants and children aged 1 to 4 years are 13.9% and 9.4%, respectively. *Shigella* uses unique effector proteins to modulate intracellular pathways. *Shigella* cannot invade epithelial cells on the apical site; therefore, it needs to pass epithelium through other cells rather than the epithelial cell. After passing epithelium, macrophage swallows *Shigella,* and the latter should prepare itself to exhibit at least two types of responses: (I) escaping phagocyte and (II) mediating invasion of and injury to the recurrent PMN. The presence of PMN and invitation to a greater degree resulted in gut membrane injuries and greater bacterial penetration. Infiltration of *Shigella* to the basolateral space mediates (A) cell attachment, (B) cell entry, (C) evasion of autophagy recognition, (D) vacuole formation and and vacuole rapture, (E) intracellular life, (F) Shiga toxin, and (G) immune response. In this review, an attempt is made to explain the role of each factor in *Shigella* infection.

## Introduction

Diarrhea is a significant public health problem that is caused by different gastrointestinal pathogens, including protozoa, viruses, and bacteria [1]. Etiologic diagnosis of infectious diarrhea is challenging due to (1) high similarity in clinical signs and symptoms and (2) having similar transmission routes [2]. Gastrointestinal pathogens are transmitted in different ways including (1) consumption of contaminated food or water, (2) person-to-person contact, (3) swimming pools, (4) exposure to animals, (5) flies, and (6) acquiring from the environment [3].

The frequency of protozoa, viruses, and bacteria varies among patients with diarrhea. Among hospitalized diarrheal patients during 2004–2006 in the Republic of Korea, the frequency of bacteria, viruses, and protozoa was 1797, 1759, and 129 per 10,000 individuals, respectively [4]. Moreover, in another published study, the frequency of protozoa, viruses, and bacteria was 20.8%, 19.6%, and 2.8%, respectively [5].

Many bacterial pathogens can be transmitted through food products such as *Shigella* spp., *Salmonella* spp., *Yersinia enterocolitica,* and *Campylobacter* spp. One of the important pathogens that causes dysentery is *Shigella* spp. [6]. *Shigella* is a gram-negative, nonmotile Enterobacteriaceae which is separated from *Escherichia coli* [7, 8]*.*

Human and other primates along with monkeys, rabbits, calves, fish, chickens, and piglets are considered as the natural hosts of *Shigella* [9]. Several different factors including raw meat consumption, unhygienic food handling practices, extensive field slaughtering practices, and unsafe water supply can affect the incidence of *Shigella* species [10].

Globally, the annual incidence rate of *Shigella* infections is estimated at 80 to 165 million cases [10]. Moreover, it is estimated that nearly 95% of all cases were related to developing countries [10, 11]. *Shigella* is one of the main leading causes of death in children under 5 years old. The death rate due to *Shigella* varied from 12 million in 1990 to 6.9 million in 2011 [12]. This pathogen accounts for approximately 11% of all deaths among children under 5 years. Moreover, the case-fatality rates of *Shigella* among infants and children aged 1 to 4 years old are 13.9% and 9.4%, respectively [12].

In most cases, *Shigella* is related to bloody diarrhea (dysentery). Based on the World Health Organization (WHO) recommendation, ciprofloxacin or azithromycin, pivmecillinam, and ceftriaxone are suitable antibiotics for the treatment of dysentery [13].

Although several live attenuated, inactivated, and subunit vaccines are available to prevent *Shigella* infections, WHO highlighted the need for novel interventions and development of new *Shigella* vaccines and antibiotics [14].

Regarding *Shigella* pathogenesis, after internalization inside the intestine lumen, bacteria should be infiltrated to the subcellular position. *Shigella* needs an M cell to cross the epithelial layer; an M cell is a particular epithelial cell that carries sampling antigen and transports it across the epithelial cell to the M cell pocket [15]. In the M cell pocket, bacteria are delivered to the resident macrophage and T cell to propagate immune responses. Following the internalization of *Shigella* into the macrophage, it massively duplicates, resulting in macrophage dying and bacterial release [16]. After release from macrophage, *Shigella* appears on a basolateral surface, and after binding with the epithelial cell, it inserts effector proteins via *type-three secretion system* (T3SS).

In general, there are several different classes of secretion systems in gram-negative bacteria (types 1 to 6), and each system transports a specific subset of proteins. The structures and mechanistic functions of secretion systems vary [17]. Gram-negative bacteria with type-1 secretion systems (T1SSs) transport their substrates including digestive enzymes, proteins with repeats-in-toxins (RTX) motifs, adhesins, and heme-binding proteins in a one-step process across both of the inner and outer bacterial membranes. T1SSs are highly similar to a large family of ATP-binding cassette (ABC) transporters [18, 19]. Similar to T1SSs, type-II secretion systems (T2SSs) have been found in a large number of gram-negative bacteria. Transporting of folded proteins from the periplasm into an extracellular environment occurs by T2SSs [20]. Type-III secretions systems (T3SSs), described as “injectisomes” and “needle and syringe,” are present in various gram-negative bacteria. T3SSs transport different proteinaceous substrates (generically called effector proteins) in a one-step process across both the inner and outer bacterial membranes. Moreover, this system can transport effector proteins into a target eukaryotic cell membrane in a one-step process [21, 22]. Type-IV secretion systems (T4SSs) are extensively found in gram-negative bacteria, and they are associated with bacterial DNA conjugation systems. T4SSs can transport single proteins, protein-protein, and DNA-protein complexes across both the inner and outer membranes [20].

In *Shigella*, T3SSs mediate internalization into the epithelial cell after engulfment with the vacuole. Then, a unique effector protein degrades double-layer vacuole, which in turn helps bacteria escape into the cytoplasm. After reaching the cytosol, *Shigella* uses an actin filament to make movements. Free movement affects the cytoplasmic membrane and makes a pseudopod; afterward, this pseudopod is swallowed by an adjacent cell [6]. *Shigella* spp. effector proteins with their mechanisms, targets, and outcomes are shown in Table 1. Overall, the infiltration of *Shigella* to the basolateral space mediates seven steps including (A) cell attachment, (B) cell entry, (C) evasion of autophagy recognition, (D) vacuole formation and vacuole rapture, (E) intracellular life, (F) Shiga toxin, and (G) immune response. In general, *Shigella* utilizes these seven steps and effector proteins to invade hosts, damage tissue sites, and thwart the immune system from responding. In this review, an attempt is made to explain the role of each factor in *Shigella* infection.

**Table 1:** *Shigella* effector proteins with their mechanisms, targets, and outcomes

Effector protein	Role	Target	Outcome
Pic	Serine protease	O-linked glycan	Impair PMN chemotaxis
IpaA	Filopodial formation	Vinculin and talin	Stimulate filopodial formation
IpaB	Formation of ion channel	Plasma membrane	Detection of host cell cholesterol
IpaB-IpaC	Pore formation	Lipid raft	
IpaD	Assembly	IpaB-IpaC (bacteria)	Cholesterol sensor, activate T3SS
	Induce apoptosis	B cell	Connection to the TLR-2 and induce apoptosis
IcsA	Induce actin polymerization	Cdc-42	Moving through actin polymerization
IcsB	Inhibit autophagy	Toca-1	Blocking recruitment of LC3 to the bacterial surface
IpgB1	Guanine nucleotide exchange factor	Rac1	Mediate activation of ELMO and formation of ELMO-Dock180 complex
IpgB2	Guanine nucleotide exchange factor	Rho	Conformations change in Rho, mimicking Dbl family
IpgD	Phosphoinositide phosphatase activity	PIP2	Produce PIP5, mediating more bacterial invasion
SpeG	Spermidine acetyltransferase	spermidine	Protects *Shigella* from ROS
IpaH 9.8	Ubiquitin E3 ligase	NEMO	Proteasomal degradation of NEMO and NF-κB activation
IpaH7.8	E3 ligase	GLMN	Activated inflammasome
IpaH4.5	Ubiquitin ligase	TBK1	Activated INF regulatory factor 3
IpaH1.4	E3 ligase	LUBAC	Catalize functional subunit of LUBAC
IpaJ	Cysteine protease	ARF1	Cleavage of myristoyl group from GTP-active protein
OspB	Remodeling of chromatin	P38-ERK1/2	Diminish inflammatory cytokine production
OspC1	Mediate activation of kinase	MEK/ERK	Imbalance membrane stability
OspC3	Mediate inhibit activation of caspase	Caspase-4	Inhibit epithelial cell death
OspF	Phosphatase activity	MAPK	Suppress gene expression
OspG	Kinase	IϏB	Inhibit activation of NF-κB
OspI	Glutamine deaminase	UBC13	Reduce inflammatory response by suppressing signaling through UBC13-TRAF6
OspZ	Methyltransferase activity	TAB-3	Inhibit signaling through TLR, IL-1

### Cell attachment

The first barrier to microbial infection is mucin glycoprotein. *Shigella* can glycosylate and remodel the mucus barrier to its benefit. Mucin is categorized into three types, including cell surface mucin, non-oligomeric gel-forming mucin, and oligomeric gel-forming mucus [23]. Gel-forming mucin as a major component of mucus can be induced by proinflammatory cytokines such as *interferon-gamma* (IFN-γ), *tumor necrosis factor-α* (TNF-α), neutrophil elastase, and microbial product [24, 25]. Stimulation of a specific gel-forming mucin namely Mu5Ac by *Shigella flexneri* (*S. flexneri*) leads to the accumulation of a gel-like structure on the apical surface that facilitates access to *S. flexneri*, thus leading to invasion. However, *S. flexneri* attempts to reduce the secretion of gel-forming mucus to reduce its distance from the epithelial cell. Nevertheless, *S. flexneri* can modify the glycosylation of mucin, resulting in changing the structure to its benefit. This phenomenon is T3SS dependent, and the MxiD mutant that lacks T3SS efficiency does not exhibit this phenomenon [26]. Given that *Shigella* cannot invade the apical epithelial surface, the target M cells penetrate the epithelial barrier. M cells are particular cells in mucosal-associated lymphoid tissue (MALT) that play a significant role in the transport of antigen from lumen to antigen-presenting cells (APC). M cells with their thin microvilli along with the absence of surface glycoprotein are exposed to the *Shigella* invasion [27]. The entrance of *S. flexneri* to the M cells leads to the recurrence of polymorphonuclear neutrophils (PMN) and increase in the size of M cell. *S. flexneri* cannot invade the apical membrane of the colonic cell; however, the recurrence of PMN leads to the destruction of epithelial conjunction and helps *Shigella* reach the basolateral space [28, 29]. Inflammation, which is induced by PMN, leads to greater permeability and further passage of *S. flexneri*. Thus, *S. flexneri* can pass through the epithelial cell by M cells or directly through permeability induced by PMN. After this, the bacteria swallowed by dendritic cells (DCs) and macrophages in the pocket of M cells degrade; however, *S. flexneri* can induce apoptosis in this APC and release itself [30]. In the first step, *S. flexneri* ruptures the vacuole and escapes to the cytoplasm; then, it fully duplicates in the cytoplasm. In the final step, *Shigella* induces host cell apoptosis to make itself free. Mucosal inflammation is partly induced by peptidoglycan and sensed by the nucleotide-binding oligomerization domain-1 (NOD1). Being a cytosolic pattern recognition molecule, NOD1 can bind with the peptidoglycan in the cell wall structure and mediate cascade signaling, leading to the stimulation of inflammatory responses. NOD1 specifically recognizes *gamma*-*D*-*glutamyl*-*meso*-*diaminopimelic acid* in the peptidoglycan of gram-negative bacteria.

After sensing this ligand by NOD1, its adaptor receptor-interacting serine/threonine-protein kinase 2 (RIP2) recurs and is finally phosphorylated [31]. This phosphorylation leads to the activation of the tumor growth factor-β (TGF-β) activating kinase 1 (TAK1). This cascade leads to the activation IKK complex and is, finally, phosphorylated and causes proteasomal degradation for the NF-*κ*B inhibitor. This effect leads to the release of p50 and p65 subunits of NF-*κ*B, thus producing the antimicrobial peptide, chemokines, and cytokines [32].

Finally, this sensing leads to the activation of nuclear *factor* kappa-B (*NF-κB*); in turn, *NF-κB* leads to transcription of interleukin 8 (IL-8), which is a major factor in the recurrence of neutrophil [33]. NOD1 also leads to the upregulated expression of intracellular adhesion molecule-1 (ICAM-1) through NF-*κ*B signaling. ICAM as transmembrane glycoprotein has a receptor role in the β2 integrin of leukocytes. This upregulation leads to greater recurrence of neutrophils to the infection site [34].

Intestinal epithelium plays a protective role in the luminal barrier and inhibits the penetration of pathogen and nonpathogenic bacteria. However, the basolateral surface is a clean area that does not encounter *lipopolysaccharides* (LPS). Thus, presentation of *S. flexneri* in the basolateral membrane may stimulate an epithelial response [35]. LPS is a major factor in forming an interaction with epithelial cells in the basolateral position, and *S. flexneri* with defects in LPS structure may affect the ability of bacteria to mediate the basolateral attachment. Attachment and recognition of LPS lead to the activation of extracellular signal-regulated kinase (ERK) and the initiation of inflammatory responses. The inflammatory response, in turn, results in PMN recurrence [36]. Invasion of apical epithelial cells is limited, which is proportional to the basolateral surface. Moreover, the addition of M cells to the apical surface mediates increased invasion by *S. flexneri* [29]. LPS and intermediate metabolite activate the inflammatory response, and tumor necrosis factor receptor (TNF-R)-associated factor (*TRAF*) protein interacting with the forkhead-associated domain (TIFA) may induce the activation of TRAF2 and TRAF6, leading to activation of the inhibitor of the nuclear factor-kB kinase (IKK). β-heptose 1,7-biphosphate as an intermediate metabolite of the LPS biosynthesis pathway mediates the activation and oligomerization of TIFA. TIFA activation prompts proinflammatory gene expression. Interestingly, β-heptose 1,7-bisphosphate causes a delay in TIFA activation and should be processed intracellularly to induce inflammation. As a metabolite, ADP heptose can mediate rapid TIFA activation and recognize new pathogen-associated molecular patterns (*PAMPs*) [37]. Although NOD-1 can detect *S. flexneri* invasion, NOD-1 facilitates detecting the early phase of the invasion. However, TIFA performs the next or final recognition phase for *Shigella* invasion through bacterial replication sensing [38]. On the other hand, the O-antigen of LPS interacts with gangliosides on the T-cell surface to mediate injection of the T3SS effector; this injection needs actin polymerization [39]. *Shigella* can only inject effector protein inside the lymphocyte without invasion, a phenomenon that used to be called injection-only. Many immune cells can be affected by injection-only such as B cell, T CD4+, T CD8+, and memory B cells [40]. Although TLR4-MD2-CD14 mediates LPS recognition on the cell surface of the eukaryotic cell, cytosolic plant disease resistance-like protein (CARD4/NOD1) mediates cytosolic recognition of the LPS. Activation and oligomerization of CARD4 by LPS of invasive *Shigella* sufficiently induce NF-κ*B* and *c-Jun N-terminal kinases* (*JNKs*) activation [35]. This activation involves not only the JNKs kinase activity but also the c-Jun phosphorylation, which finally leads to the regulation of inflammatory response [41]. LPS of *Shigella* is hexa-acylated and can be modified when the growth of bacteria inside the epithelial cell occurs. This modification made by hypoacetylation provides a chance for inducing an immune response, reducing the capacity to induce oxidative burst by the PMN, and downregulating the release of IL-1β from the infected macrophage [42]. Altogether, the response released by *Shigella* facilitates survival, in all probability.

#### IcsA (VirG)

Attachment is the beginning step in infection, which is the most important step in the beginning of pathogenesis. The *S. flexneri IcsA* (VirG) protein is sufficient for the attachment process that helps bacteria spread through actin polymerization inside the cell [43]. Ten amino acid regions (138–148) are required from IcsA to ensure adhesion to the host cell. IcsA triggers signal transduction in the N-terminal which mediates secretion through the autotransporter system. The N-terminal region provides a chance for translocation across the inner membrane, while the β-barrel domain facilitates translocation through the outer membrane [44]. Furthermore, IcsA has a polar targeting region that mediates attachment to the old pole of the bacterial cell. Passenger-associated transport repeat (PATR) with conserved glycine residue has a significant role in the transporting and surface exposure of the IcsA. IcsA is secreted through the Sec system and polarized on the pole side of the bacterial cell. In addition, it can be secreted from the bacterial cell surface with the aid of IcsA protease (IcsP). Therefore, the signal peptide of IcsA has two distinct residues that mediate two distinct conformations; first, it is attached to the pole side of bacteria, while the latter mediates secretion into the environment [45]. IcsP causes catalytic activities through protease measures against IcsA accumulated in the non-pole region. The localization of IcsA occurs through the instrumentality of a specific protein PhoN2 which is strictly found on the pole side of the bacterial cells [46].

This adhesin can be activated by sensing bile salt through the T3SS. Bile can stimulate *S. flexneri* to attach to and invade the epithelial cells. In addition, it enhances the expression of the *Shigella* effectors ospE1/ospE2 as adhesins [47, 48]. OspE can interact with integrin-like kinase to fix cell adhesins, and this interaction increases the integrin level. Therefore, upon increasing the level of integrin, *Shigella* inhibits detachment and shedding of infected cells [49]. The exposure of *S. flexneri* to bile for a long time can induce biofilm formation. Bile sensitivity is dose dependent; in this way, through the reduction of bile in the ileum, biofilm dispersion leads to the invasion of the colon by *S. flexneri* [50, 51]. *S. flexneri* can subvert cell division control protein-42 (Cdc-42) via IcsA and finally cause actin polymerization. First, IcsA binds with the neural Wiskott-Aldrich syndrome protein (N-WASP); then, N-WASP mediates the recurrence of Arp2/3, thus making IcsA-N-WASP-Arp2/3 complex [52]. These complexes induce actin polymerization. Region A (acidic motif) of WASP binds with Arp2/3, and consequently, actin monomer is added to the actin filament [53]. In a natural state, the Cdc42-N-WASP-Arp2/3 complex keeps actin polymerization in check [54]. IcsA greatly mimics Cdc42 to help bacteria bind the N-WASP to Arp2/3 [52]. Interestingly, the special N-terminal of IcsA only forms a connection with the calmodulin-binding IQ motif of N-WASP; this finding makes it clear as to why *Shigella* only induces actin rearrangement in N-WASP expressing cells such as an epithelial cell [55, 56].

#### Pic (protease involved in colonization)

Pic belongs to class 2 of serine protease autotransporters of Enterobacteriaceae (SPATE), and it is found in enteroaggregative *Escherichia coli* and *S. flexneri* [57, 58]. The O-linked glycan can affect the Pic in leukocyte adhesion proteins such as CD45, CD44, CD43, CD93, and P selectin. This adhesion is involved in migration and cell trafficking. O-linked glycan is clustered in the mucin-like domain protein, which was influenced by Pic [58, 59]. Therefore, this cleavage by Pic affects PMN chemotaxis and migration. However, Pic via mucinase activity helps *Shigella* penetrate the mucus layer [60]. It also makes a distinction between the coagulation factor V and pepsin A [61]. Altogether, Pic may mediate the growth and intestinal colonization of *Shigella* by serine protease activity against mucin [62].

### Cell entry

#### Cell entry

T3SS is encoded via a large plasmid and has been used to translocate the effector protein to the eukaryotic cell. The attachment component of T3SS consists of IpaB, IpaC, and IpaD members. The first two members are involved in the formation of pores in the eukaryotic cell, and the latter facilitates the assembly of the first two members [63]. IpaD is a hydrophilic protein that binds with the tips of T3SS through C-terminal and is composed of four subunits that block the needle pore in order to inhibit secretion. A critical step in initial binding depends on sphingolipid, and the cholesterol-rich domain is called lipid raft. After depletion of cholesterol, *the S. flexneri* invasion is impaired [64]. First, IpaD should sense the existence of cholesterol and sphingomyelin to stimulate invasion against plasmid antigen B (IpaB) to be present in the T3SS tip. However, sensing bile via IpaD can promote IpaB exposure at the tip [65]. Direct interaction between deoxycholate and IpaD mediates the recruitment of IpaB to the T3SS tip. After this interaction, a conformational change is made to the IpaD which leads to the recruitment of IpaB to the T3SS tip [66].

Interaction between IpaB and IpaD at the tip is a significant factor in the host cell sensing and T3SS activation [63, 67]. Following the formation of the connection between IpaB and the host cholesterol, T3SS was activated to inject effector protein into the host cell. Finally, after introducing IpaB to the tip of T3SS, IpaC recurs to the tip, and the T3SS is activated [68].

Following the activation of T3SS, IpaB, IpaC, and IpaD are released, and they bind with the α5β1 integrin, thus mediating actin rearrangement.

IpaB can act as an ion channel in the host cell membrane that leads to the influx of potassium (K+), which is recognized via NLR family CARD domain-containing protein 4 (NLRC4) resulting in the activation of pyroptosis. Potassium plays an essential role in membrane stability; thus, the imbalance between potassium ions intensifies osmotic pressure and vacuole rapture.

Interestingly, the reduction of intracellular K+ concentration stimulates NLRP3 (NOD-, LRR-, and pyrin domain-containing protein 3) [69]. This molecule causes host cell invasion and phagosome escaping [70]. IpaB can directly bind with the hyaluronan receptor CD44 that is located in the basolateral membrane of the cell and participates in bacterial invasion [71]. Finally, IpaC may cause the activation and return of Src tyrosine kinase, leading to actin polymerization at the bacterial entry [72]. After actin rearrangement, *Shigella* enters the epithelial cell. Src is translocated to the plasma membrane and attached to the inner surface; Src activities lead to signal transduction [73]. After this signaling, *Shigella* is swallowed in the vacuole and should activate the T3SS. IpaC is a chaperone attached to the IpaB in the bacterial cytoplasm and is separated before secretion in the extracellular milieu [74, 75]. Furthermore, following the engulfment of bacteria inside the vacuole of epithelial cell, T3SS helps bacteria evade the vacuole and release it to the cytoplasm [76].

#### B-cell infection

The interaction between the IpaD and Toll-like receptor 2 (TLR-2) leads to mitochondrial apoptosis in B cells. Interestingly, this interaction needs a bacterial co-signal to sensitize B cells and to upregulate the TLR-2; the upregulation of the TLR-2 mediates the attachment of IpaD to TLR-2 and triggers the apoptosis [77]. Infection with wild-type *Shigella* that possesses a T3SS leads to the death of B cell in both invaded and non-invaded B lymphocytes.

Signaling by TLR-1 mediates apoptosis, and it is indicated that TLR-1/2 heterodimer generates death signal by IpaD. Altogether, *Shigella* has the power to invade the B lymphocyte and proliferate inside it, leading to cell death [77].

##### IpaA

Vinculin and talin as mediate cell adhesins have a distinct role. Talin as an integrin-associated protein (IAP) mediates the adhesion of integrin to the extracellular matrix and links integrin directly to the actin cytoskeleton. Talin increases focal adhesion, senses matrix rigidity, and is a platform for adhesin structure [78, 79]. Recurrence of talin to the site of ligand-bound integrin mediates the talin connection between integrin and actin to make the integrin-talin-actin complex; this complex mediates filopodial formation [80]. IpaA can bind with vinculin and talin, leading to the formation of filopodial adhesin and capturing of *Shigella*. IpaA contains vinculin binding sites (VBS) and binds with talin to make filopodial adhesin [81]. After the attachment of IpaA to talin, talin semi-stretches and stimulates *Shigella* capturing. The C-terminal of IpaA VBS by mimicking the activity of talin VBS modulates the function of vinculin to be recruited to the bacterial entry [82].

### Evasion of autophagy recognition

#### IcsB

Autophagy is a mechanism that degrades macromolecule in the cytoplasm to recycle energy and damaged organelles, and it consists of assembling many autophagy-related proteins (ATG). One important form in canonical autophagy is the formation of the double-membrane enclosure, a so-called phagophore that finally converts into the autophagosome [83]. Accumulation of microtubule-associated protein light chain 3 (LC3) and ATG16L1 causes the maturity of phagophore to the autophagosome. In bacterial cases, a bacterial component including peptidoglycan can be sensed by NOD that restricts bacterial survival. NOD causes the recurrence of ATG16L1 to the invasion site and autophagy activation [84]. In noncanonical autophagy, ATG proteins, such as LC3-associated phagocytosis (LAP), can be activated, and they recur to the already damaged membrane vacuole [85]. Following the activation of the T3SS, in noncanonical autophagy, the LAP can be activated; however, IcsB as an IpgA chaperone has an influential role during cell-to-cell spread [86]. Following the invitation of the transducer of Cdc42-dependent actin assembly 1 (Toca-1), IcsB can block the recurrence of LC3 to the bacterial surface [87]. Toca-1 is required for bacterial spread through the actin tail. However, for actin rearrangement, both N-WASP and Toca-1 are required. N-WASP should be activated by Toca-1 for Cdc-42 activation and actin polymerization [88] (see the “Actin rearrangement” section). After the degradation of the vacuole and its release, IcsA can be recognized by the ATG5; moreover, ATG5 can interact with tectonic beta-propeller repeat-containing 1 (TECPR1) protein. TECPR1 interacts with PI3P to induce LC3 and activate autophagy [89]. Following the attachment of TECPR1 to the PI3P, in the next step, it is attached to the ATG5 and localized in the autophagosome targeting bacteria. However, IcsB masks the region that is again unmasked by ATG5, thus inhibiting the recognition by LC3 [90].

### Vacuolar formation and vacuolar rupture

#### IpgD

After the internalization of *Shigella*, vacuole forms around the bacteria. IpgD effector protein is translocated via the T3SS, and this effector is a homolog to the *Salmonella* SopB. IpgD has a phosphoinositide phosphatase activity and dephosphorylates phosphatidylinositol 4,5-biphosphates (PIP2) into phosphatidylinositol 5-phosphate (PI5P) [91, 92]. PI5P can be increased through the stimulation of osmotic shock or during *S. flexneri* infection [91].

Furthermore, PI5P activates the epidermal growth factor receptor (EGFR) in a ligand-independent manner and mediates the recurrence of the target of myb-1 (TOM1) to lagging EGFR degradation [93]. Blocking the degradation of EGFR lead to continuous signaling and cell survival. The recurrence of TOM1 as a membrane trafficking regulator by PI5P leads to the inhibition of endosomal maturation [94]. TOM1 directly binds with the PIP5 as an effector to regulate endosomal maturation. Increased PI5P level leads to the actin rearrangement and greater bacterial invasion.

Moreover, PI5P activates and phosphorylates Akt and ensures cell survival [95]. The phosphatidylinositol 4,5-bisphosphate is cytoskeleton remodeling [96] and activates Akt to mediate cells to survive. Following the internalization of *Shigella* to the vacuole, the first marker Rab5 and early endosome A1 (EEA1) stay on the surface of the vacuole and then exchange with Rab7 to mediate lysosome degradation [97]. Rab5 and early endosome transiently appear and disappear. Rab11 mediates early/recycling endosomes to endoplasmic reticulum [98]. Another role of IpgD is mediating the recurrence of Rab11 to the early vacuole-containing *Shigella*. This massive recurrence of Rab11 to the invasion site continues until the point of the vacuole explosion. The fusion of Rab11 to the nascent vesicle leads to the promotion of its rupture [99]. IpgD can block the recurrence of T lymphocyte to the infection site and evade immune response [100]. Through the production of PI5P, IpgD protects early endosomes from lysosomal degradation.

The depletion of PIP2 via IpgB can block actin formation around the vacuole containing *S. flexneri* which, in turn, results in vacuolar destabilization [93]. The production of PI5P via IpgD leads to the internalization of ICAM-1, which is a significant adhesin playing a part in the recurrence of immune cells. ICAM-1 as a leukocyte receptor can be internalized by PI5P and directed to lysosomal degradation; so, IpgD as a PI5P producer blocks leukocyte recurrence [101]. PI5P performs the internalization of the surface receptors such as EGFR and ICAM-1; however, binding between TOM1 and PI5P interestingly leads to stabilization of the EGFR on the surface. However, another effector may interact with ICAM-1 to internalize lysosome and degrade it [94, 101]. IpgD inhibits the release of ATP as an inflammatory signal from the infected epithelial cell. *S. flexneri* may open connexin 26 hemichannels and stimulate the ATP release. However, PI5P production from PIP2 by IpgD blocks the hemichannel and forces the ATP release [102]. Interestingly, at the beginning of the infection, *Shigella* stimulates the release of ATP to induce inflammation and membrane disorder which favors the mucus passage [103]. Thus, after the passage of *Shigella*, bacteria do not need inflammation and block the ATP release.

#### VirA

Being a secretory protein that acts as a GTPase activating protein (GAP), VirA can be inactivated by Rab1, and it disrupts the trafficking of endoplasmic reticulum (ER) to the Golgi [104]. Disruption of ER to Golgi trafficking blocks autophagosome formation. Furthermore, VirA can stabilize Rab1 in the deactivated form (GDP) and block autophagy [85]. After the internalization of the vacuole, VirA activity disrupts vacuole and intracellular spreading. This effector is similar to the IcsB and works together to disrupt vacuole. LC3 particularly recruits monolayer vacuole-containing bacteria and does not affect free bacteria in the cytoplasm [90].

#### T3SS effects

A special protein-containing syringe mediates the transport of effector protein and chaperon from bacterial cytoplasm to eukaryotic cytoplasm. This syringe is regulated by temperature and can only be assembled at 37 °C, a process that keeps the transcription shut down until the appropriate time [105]. Following the invasion of the epithelial cell, Ca2+ responds to the inducing of inositol 1,4,5-triphosphate (IP3). IP3 is generated by phospholipase C (PLC). Finally, IP3 is attached to the IP3 receptor at the entry site of bacteria and leads to the release of Ca2+ [106]. Calcium influx can be activated by calpain, a protease that degrades p53. Calpain remodels the cytoskeleton and helps *S. flexneri* form filopodia [107, 108]. Calpain can degrade p53 through the protease activity and induce apoptosis [109]. Increase in the value of Ca2+ leads to the opening of the connexin channel and mediates the release of ATP to the extracellular milieu. This atypical Ca2+ response at the entry site mediates cytoskeleton rearrangement and *S. flexneri* engulfment [110]. However, Pilus protein FimA inhibits the release of cytochrome C by mitochondria and intervenes in the apoptosis process [111].

Interestingly, the activation of calpain by VirA in a Ca2+ dependent manner finally leads to cell necrosis. Therefore, *Shigella* should regulate cell death at the later phase of infection, mediating opportunities to full duplication. The presence of oxygen can regulate the activation and secretion of effector proteins by T3SS. In the lumen and anaerobic conditions, the virulence gene regulates spa32 and spa33 repressed by fumarate and nitrate (FNR). However, reverse repression mediated by FNR occurs near the mucosa through the diffusion of oxygen from the tip of villi [112]. Interestingly, *Shigella* aerobic respiration causes oxygen depletion through the formation of the colonic extracellular matrix in the foci of infection. Therefore, this depletion leads to T3SS repression as a primary strategy for the beginning of colonization [113].

#### IpgB1 and IpgB2

IpgB1 and IpgB2 have guanine nucleotide exchange factor (GEF) activities that affect Ras homolog gene family, member A (RhoA), and Rac family small GTPase 1 (Rac1), leading to actin reorganization. Signaling through Rho needs to undergo a change from the GDP to GTP form [114]. IpgB1 and IpgB2 activate Rac1 and Rho, respectively. Rac1 activation leads to actin polymerization, and Rho activation causes actin-myosin contraction [115]. Interestingly, the activation of Rho leads to Rac downregulation. The entrance of *Shigella* needs the membrane ruffling, and this activity needs the RhoG-ELMO-Dock180 complex to induce Rac1. IpgB1 mimics the role of RhoG and binds with the engulfment and motility protein (ELMO), and this complex activates Rac1 [116]. Dock180 (dedicator of cytokinesis) superfamily acts as a particular guanine nucleotide exchange factor for Rho GTPase, and ELMO (the engulfment and migration protein) is a regulator for Dock180. In fact, after activation of ELMO by IpgB1, the ELMO-dock180 complex recurs to the membrane plasma and activates Rac1 [116, 117]. Finally, GEF activity prompts the IpgB to recur to the state of membrane ruffling and induces bacterial internalization to the bacterial-containing vacuole (BCV) [118]. IpgB2 can activate the RhoA pathway by mimicking the function of RhoA, leading to the activation of RhoA effector Rock and mDia [119]. By mimicking Dbl activity, IpgB2 which is a multifunctional molecule plays the role of GEF and mediates Rho activation [120]. Furthermore, IpgB2 can activate NOD1 after an attack on the epithelial cell and finally activate *NF-κB*. Interestingly, GEF-H1 of host cells mediates RhoA activation and interacts with NOD1, leading to *NF-κB* activation. GEF-H1, which is a significant factor in the activation of the *NF-κB*, can interact with NOD1 [121]. In a reasonable condition, GEF-H1 connects to cingulin; however, after invasion by *Shigella*, it is released and transfered to connect to NOD1. This connection mediates NOD1 signaling and finally causes *NF-κB* activation. This mechanism is working through the detection of peptidoglycan components and is independent of the GEF activity. NOD1, in addition to the recognition of peptidoglycan, together with GEF-H1, can detect *Shigella* effector proteins [121, 122].

#### SpeG

Polyamines including spermine and spermidine are involved in many processes such as survival, cell growth, gene expression, biosynthesis of siderophores, free radical ion scavenger, and acid resistance [122, 123]. Spermidine provides protection against oxidative stress, reduces *nuclear translocation* of *NF*-*κB p65 subunit*, and decreases the quantity of LPS-induced *reactive oxygen species* (ROS) [124]. SpeG as a spermidine acetyltransferase converts spermidine into acetylspermidine, which is not functional. However, evolutionary silencing of this gene in *Shigella* accumulates spermidine inside the bacteria. Therefore, this accumulation enhances the survival chance for *Shigella* against oxidative stress and leads to free radical scavenging [125].

### Intracellular life

After the multiplication of *Shigella* in a host cell, damage and inflammation occur. One strategy to terminate intracellular bacterial life is to program cell death. Recognition of components such as LPS and T3SS via nod-like receptor (NLR) and TLRs leads to inflammatory caspase activation [126]. Furthermore, the LPS recognition triggers caspase-4 and caspase-11 to activate pyroptosis, resulting in cell death and intestinal epithelial cell shedding [127]. Pyroptosis is activated through NLR and mediated by IL-18 and IL-1β, resulting in membrane rupture. Membrane rupture in the case of pyroptosis cells by caspase-1 leads to ion venting and inflammatory response [128]. The lipid-A component of cytoplasmic LPS can directly bind with the caspase-11, resulting in the activation of the inflammasome and pyroptosis [129]. However, *Shigella* can prevent epithelial cell death before total duplication [130]. Cell death also is induced through mitochondrial injury regulated through the interaction between cyclophilin D and Bcl-2/19kDa protein 3 (Bnip3). NOD1 detects bacterial components and causes protection against the activation of Bnip3 and cyclophilin D, ensuring protection against epithelial cell death. Therefore, NOD1 may mediate protection against cell death in nonmyeloid cells [131]. Protection against cell death by NOD1 depends on the ability of NOD1 to induce *NF-κB* [132]. After the entrance of *Shigella* to the epithelial cell, membrane ruffle forms around the bacteria and leads to the recurrence of NOD1 and the component of NOD1 downstream signaling NF-κB essential modulator (NEMO) to the bacterial positions. Localization of NOD1 in the plasma membrane depends on F-actin [133]. Another type of NOD1, NLRs as an immune sensor for bacterial components, consist of two parts: nucleotide-binding domain (NBD) and leucine-rich repeat (LRR). Being autoinhibitory, LRR inhibits the activation of the NBD domain and, as a sensor, directly or indirectly detects the microbial components [134, 135]. In macrophages, the activation of NLRC4 (nod-like receptor C4) and NLRP3 (nod-like receptor P3) commenced, thus inducing pyroptosis and secretion of IL-1β and IL-18 [136]. MxiH needle protein of T3SS is detected by neuronal apoptosis inhibitory protein (NAIP), leading to the activation of NLRC4 inflammasome [137]. This activation mediates the release and activation of human neuronal apoptosis inhibitory protein (hNAIP). hNAIP can sense T3SS components and flagellin and cause NLRC4 inflammasome activation. However, given that *S. flexneri* does not express flagellin, MxiH has a significant role in the activation of NLRC4 [138]. MxiH is also injected into the host cytoplasm and modulates antimicrobial gene transcription [139]. NLRC4 can detect conserved T3SS components and distinguish between T3SS-positive and T3SS-negative bacteria [136]. However, recognition of and response to MxiH can be done in a dose-dependent manner (in low doses), thus leading to the activation of caspase and pyroptosis. However, in high doses, activated NLRP3 leads to pyronecrosis [138]. Pyronecrosis is considered as a subtype of necrosis and is a caspase-independent cell death pathway. Pyronecrosis is activated through the NAIP-dependent pathway, and this activation is induced by the mutation of the NAIP gene or microbial pathogens. Therefore, altogether, *Shigella* may trigger cell death through both apoptosis and necrosis [140]. It should be noticed that almost in early events, the caspase-1-dependent mechanism mediates apoptosis; however, in later events, caspase-1-independent apoptosis occurs by lipid A [141]. This event explains that at the initial phase of the infection, whose bacterial dose is low, caspase-dependent death occurs; however, after the replication, caspase-independent apoptosis occurs with a considerable amount of lipid A. NAIP2 as an NLR family can directly bind with the T3SS rod proteins and induce caspase-1 activation. Upon binding with its ligand, NAIPs with NLRC4 formed an inflammasome. NAIP2, as an immune sensor, regulates the oligomerization of NLRC4 and the formation of the NAIP2-NLRC4 complex [142]. As a homolog to the NAIP1 in the mouse model, hNAIP can recognize the T3SS needle MxiH and activate NLRC4 inflammasome [138, 143]. Interestingly, in the intracellular life of *Shigella*, the T3SS is dampened but reactivated during actin-based motility and cell-cell spread [144] (see “IpaH family” section).

#### IpaH family

IpaH is encoded via both chromosome and plasmid; however, IpaH gene in the chromosome interestingly plays no role in pathogenesis [145]. IpaH has a C-terminal via catalytic activities toward ubiquitin and N-terminal leucine-rich repeat (LRR). LRR can be sensed through a pathogen-associated molecular pattern (PAMP) of the host cell [146]. This effector enters the host cell in a T3SS-independent manner and is internalized via the endocytic mechanism [147]. Ubiquitylation of protein is involved in many cellular processes including cell cycle, protein degradation, endocytosis, and inflammatory response [148]. Ubiquitylation involves three enzymes: E1 as a ubiquitin-activation enzyme, E2 as a ubiquitin-conjugating enzyme, and E3 as a ubiquitin ligase [148]. After escaping *Shigella* from the vacuole, the damaged vacuole membrane can be sensed by ubiquitin. Ubiquitinated proteins attract adaptor p62 and autophagy markers such as LC3 [149]. LC3 detects and binds with the leftover of the damaged vacuole membrane. p62, as a scaffolding protein, can interact with the ubiquitin-associated domain of tumor necrosis factor receptor-associated factor 6 (TRAF6). p62 directly binds with the autophagic protein LC3 and ubiquitin via N-terminal and C-terminal, respectively [150]. p62 leads to polyubiquitination of TRAF6, and in turn, TRAF6 causes the activation of NF-κB [151]. Activation of NF-κB yields many activities such as innate immune response, cell survival, and inflammatory response in the cell [152]. In a normal cell, NF-κB binds with its inhibitor, i.e., an inhibitor of κB (IκB), but after signal stimulation, IκB kinase is activated. IκB kinase is composed of an NF-κB essential modulator (NEMO), IKK1, and IKK2 [153]. After the activation of IKK, proteasome can cause the degradation of IκB and the release of NF-κB [154]. Ubiquitin ligase activity of TRAF6 mediates the activation of NF-κB and IKK. In addition, surprisingly, TRAF6 as an E3 ubiquitin ligase can ubiquitinate NEMO [155]. This ubiquitination leads to the recurrence of IKK and the initialization of signaling. Activation of IKK results in the phosphorylation of IκB and polyubiquitination, in turn leading to the proteasome degradation of IκB and the release of NF-κB to the nucleus [156].

##### IpaH9.8

Following the escape of *Shigella* from bacteria-containing vacuole, it can freely move inside the cytoplasm until IFN-Ƴ induces guanylate-binding protein (GBP). *Cytosolic bacteria* can be trapped by GBP. Interestingly, IpaH9.8, as a ligase, binds with GBP and ubiquitinates it. Therefore, IpaH9.8 exposes GBP to degradation by the proteasome [157]. Furthermore, IpaH9.8, which is a ubiquitin E3 ligase, affects NEMO and causes proteasome *polyubiquitylation* of NEMO, proteasomal degradation, and suppressed activation of NF-κB [158].

##### IpaH7.8

The inflammasome can sense abnormality in the cell such as lysosomal rupture, Ca2+ signaling, and mitochondrial damage. Inflammasome activation leads to caspase-1 activation which finally leads to the secretion of IL-18 and IL-1β as well as pyroptosis [159]. IpaH7.8 via E3 ligase activity has a vital role in activating inflammasome by NLRC4 and NLRP3 in a dependent manner. Glomalin/flagellar-associated protein 68 (GLMN), a member of the negative regulator of NLR inflammasome, acts as an E3 ligase inhibitor. Glomalin binds with RBX1 and masks the E2 binding site to inhibit the E3 ligase activity of RBX1 [160]. E3 ligase activity of RBX1 mediates proteasomal degradation of ubiquitinated proteins. However, GLMN in the presence of IpaH7.8 through E3 ligase activity is polyubiquitinated and enzymatically degraded to finally activate RBX1, thus leading to pyroptosis [161].

##### IpaH1.4

IpaH1.4 as an E3 ligase is another cell protector. The E3 *ubiquitin* ligase *complex and linear ubiquitin chain assembly complex* (*LUBAC*) have an antibacterial mechanism and are recruited on the bacterial surface that already has a ubiquitin. As an E3 ligase, IpaH 1.4 can interact with LUBAC and catalyze the functional subunit [162]. The GLMN binds with the inhibitor of apoptosis (IAP), a member of E3 ligase, and causes a reduction in the ligase activity; thus, the reduction of the E3 ligase activity of the GLMN by IpaH7.8 enhances inflammasome activation and forces pyroptotic cell death [163].

##### IpaH4.5

Another IpaH member, IpaH4.5, can interact with Tank-binding kinase 1 (TBK1) through ubiquitin ligase activities. As an INF regulator, TBK1s activate INF regulatory factor 3 (IRF3) which mediates INF activation. Upon polyubiquitination of TBK1, IpaH4.5 causes proteasome degradation and inhibits IRF3 activation. Altogether, IpaH4.5 inhibits INF activation and cytokine expression, thus dampening the antibacterial response [164]. In a normal cell, proteasome regulatory particle non-ATPase 13 (RPN13), a component of the 19S proteasome that acts as a regulatory subunit on the 26S, mediates ATP-dependent degradation of ubiquitinated protein. Through E3 ubiquitin ligase activities, IpaH4.5 targets and degrades RPN13 results by inhibiting 26S proteasome activities. This outcome leads to the suppression of proteasome-catalyzed peptide and reduction of cross-presentation to CD8+ cell [165].

#### IpaJ

IpaJ is separable from the myristoyl group of GTP-active protein through cysteine protease activities. However, IpaJ is characterized by specificity to the ADP-ribosylation factor 1 (ARF1). ARF1 is found in the Golgi membrane, mediates vesicle trafficking, and regulates vesicle formation as well as ER-Golgi transportation [166, 167]. ARF1 facilitates the recurrence of coat proteins to the Golgi and has an essential role in the secretory pathway [168]. IpaJ separates the myristoyl group from ARF1 and inhibits vesicular trafficking [167]. In addition, it inhibits the activation of STING, a protein associated with ER membrane, and mediates the sensing of pathogens. The STING activates IFN-I through TBK1 signaling. By blocking the STING translocation from ER to the ER-Golgi intermediate component (ERGIC), IpaJ can inhibit the activation of INF-I [169].

#### OspB

**Fig. 1:**
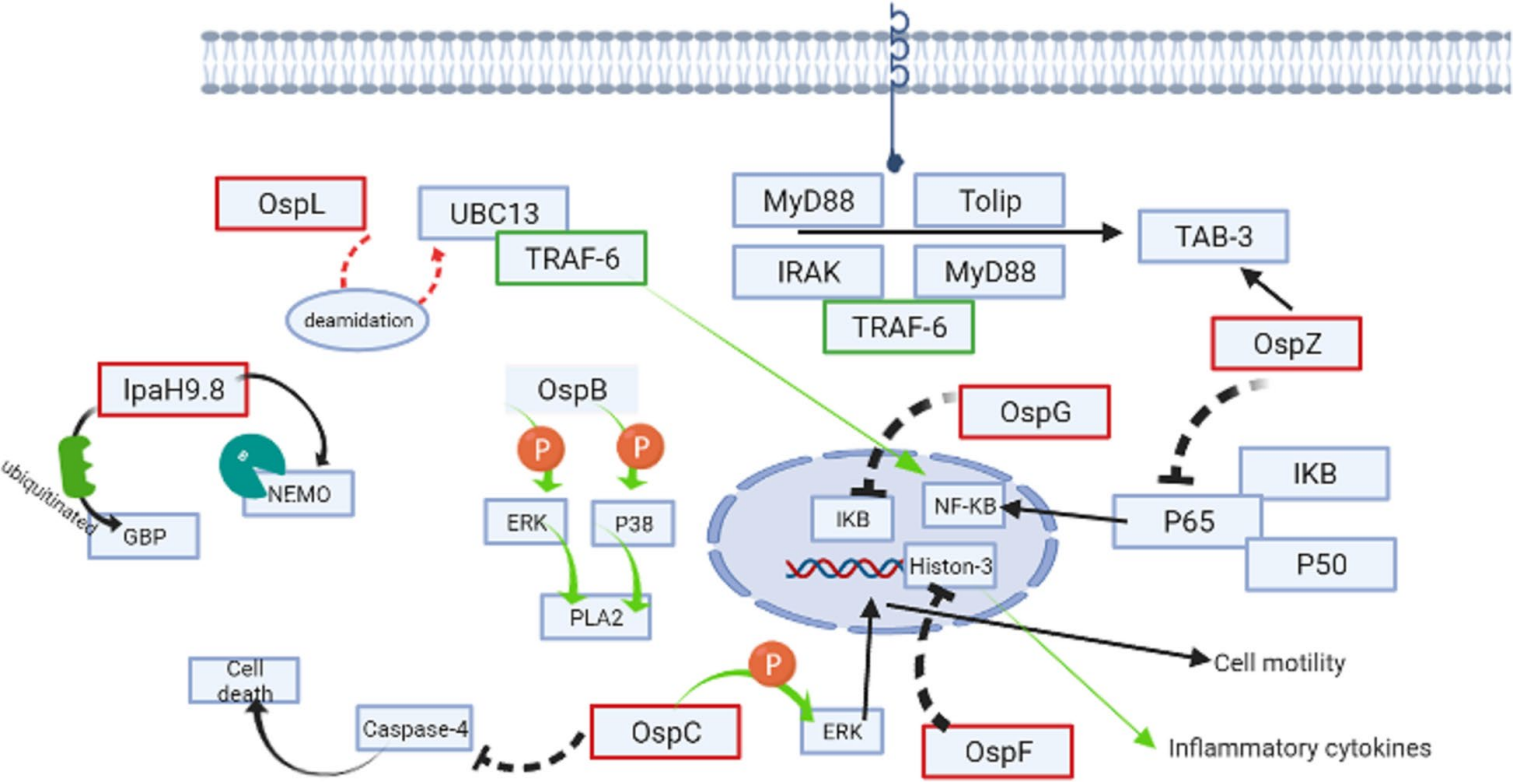
Interaction between *Shigella* effectors and the host cell that mediated activation and suppressive effects. OspI, *Shigella* effector encoded by ORF169b on the large plasmid and delivered by the T3SS; Ubc13, ubiquitin-conjugating enzyme that has a main role in the NF-κB signal transduction pathway in human infections; TRAF, tumor necrosis factor receptor (TNF-R)-associated factor; IpaH, invasion plasmid antigen H *gene* sequence; NEMO, NF-κB essential modulator; GPB, guanylate-binding protein; OspB, effector that is secreted through the T3SS; ERK, extracellular regulated kinase; PLA2, phospholipase A2; IRAK, interleukin-1-receptor-associated kinase; MyD88, myeloid differentiation primary response 88; IKB, inhibitor of NF-*κ*B

OspB is an effector that is secreted through the T3SS and is involved in the activation of p38, ERK1/2, and phospholipase A2 (PLA2). Activation of phospholipase A2 leads to the secretion of chemoattractant and IL-8 [106]. Immediately after secretion, OspB can stimulate phosphorylation of ERK1/2 and p38 [170]. Activation of p38 and ERK1/2 leads to the activation of PLA2 which mediates eicosanoid generation, eicosanoid involved in immune responses such as inflammation, and recurrence of PMN to the infection site [171]. OspB also interacts with the mechanistic target of rapamycin complex 1 (mTORC1) to facilitate cell proliferation. The activity of mTORC1 as a master regulator of cell growth and proliferation depends on the IQ motif containing GTPase activating protein 1 (IQGAP1). IQGAP1 plays a significant role in the assembly of actin and is affected by OspB directly. It also mediates mTORC1 activation. Finally, this activation and cell growth mediate niche protection inside the cell [172] (Fig. 1).

#### OspC1/OspC3

OspC1 mediates the activation of MEK/ERK and phosphorylation of ERK1/2, thus leading to the translocation of ERK to the nucleus and phosphorylation and also activation of the transcription factor. Crucial roles of the ERK include cell motility and cell survival [173]. Therefore, *Shigella flexneri* by inducing inflammation leads to the recurrence of neutrophil and imbalances the stability of membrane which mediates access to the submucosa [174]. In the mouse model, *S. flexneri* fails to elicit IL-8 and to allow neutrophil to recur to the infection site, a phenomenon that explains mice resistance to the *Shigella* infection [175]. After infection via *Shigella*, caspase-4 mediates epithelial cell death. Interestingly, *Shigella* can inhibit the activation of Caspase-4 by OspC3. Caspase-4 has two subunits, p10 and p19, and OspC3 interacts with the subunit p19 to inhibit the activation of Caspase-4 [176]. Ankyrin repeat of OspC inhibits caspase-4 conserved in other bacteria such as *Rickettsia ricketssii* and *Legionella pneumophila* [130].

#### OspF

OspF has a phosphatase activity and interacts with MAPK signaling. OspF can interact with chromatin reader, heterochromatin protein 1Ƴ (HP1Ƴ), and Histone-3 to dephosphorylate and suppress gene expression. To activate the HP1Ƴ and Histone-3, MAPK should be phosphorylated in both proteins [177]. HP1Ƴ, as a transcription regulator, has multiple phosphorylation sites, and Serine 83 has a significant role in the process. MSK1, as an HP1Ƴ kinase, phosphorylates HP1Ƴ at Serine 83. OspF can inactivate ERK and downstream kinase MSK1 by serine 83 dephosphorylation [178]. OspF translocates to the nucleus and interacts with Histone-3 to control the expression of inflammatory cytokine. Phosphorylation of Histone-3 is necessary for chromatin availability to transcription factor NF-κB; thus, it inhibits Histone-3 phosphorylation by OspF and blocks the activation of a gene which is under the control of NF-κB [179]. OspF can interact with retinoblastoma that leads to downregulation of histone modification and mediates blocking of inflammatory cytokine production [180]. Interestingly, OspF can directly interact with HP1Ƴ and dephosphorylate it. A small ubiquitin-related modifier (SUMO) can modify OspF and mediate nuclear localization and dephosphorylation activity of OspF [181]. Nevertheless, how is OspF translocated to the nucleus? Importin-α, as a heterodimer, targets a different protein to translocate across the nuclear membrane. By binding of the nuclear signal localization (NLS) of the target protein, importin-α connects to the importin-β which mediates translocation to the nucleus. Interestingly, OspF is translocated through importin-α to the nucleus and interacts with MAPK. Through phosphothreonine lyase activities, OspF interacts with X-residue in the MAPK and degrades the threonine hydroxyl group [182, 183].

#### OspG

In normal conditions, NF-κB is suppressed by inhibitory protein (IκBs). Signaling from extracellular or intracellular cell leads to inhibitor phosphorylation and of IκB ubiquitination, leading to proteasomal degradation of inhibitor and release of NF-κB [184]. With the similarities to the eukaryotic protein kinase, OspG can inhibit not only the degradation of phosphorylated IκB but also the activation of NF-κB. In other words, OspG cannot block TNF-α signaling but may suppress the degradation of phosphorylated IκB [185].

#### OspI

As a glutamine deamidase, OspI can interact with ubiquitin-conjugating enzyme 13 (UBC13) and deamidase glutamine residue in UBC13. UBC13 is an E2 ubiquitin enzyme and a significant factor in activating NF-κB by TRAF6 signaling. OspI reduces the inflammatory response by suppressing signaling through the UBC13-TRAF6 complex [186, 187]. Conversion of glutamine into glutamate by OspI leads to the inhibition of the synthesis of polyubiquitin chain and UBC13/TRAF6, which resulted in inhibiting the activation of NF-κB. Interestingly, deamidation occurs outside the UBC13/TRAF6 interaction, but changing the salt-bridge interaction inhibits regular interaction between UBC13 and TRAF6 [188].

#### OspZ

P65, as a transcription subunit of NF-κB, should be translocated and phosphorylated toward the nucleus to mediate the expression of inflammatory cytokines. OspZ can interact with p65 and block the translocation of p65 to the nucleus. Altogether, this suggests an inhibitory mechanism that blocks activation of NF-κB [189]. Through methyltransferase activities, OspZ can interact with host adaptor protein TAK-binding proteins 3 (TAB3) that mediates signaling through IL-1 and Toll-like receptor (TLR) [190]. Cysteine residue of TAB3 mediates the link between polyubiquitin and the target protein including TRAF6. This polyubiquitination leads to the formation of a complex with IκB kinase and degradation of IκB, resulting in NF-κB signaling. OspZ modifies cysteine residue of TAB3 through methyltransferase activities and disrupts the ubiquitin-binding activities of TAB3 [191, 192]. This disruption leads to the inhibition of NF-κB response to IL-1β and TNF-α.

#### Actin rearrangement

Many cellular processes include immune response, motility, and shape rearrangement that need an actin network. A core set of proteins includes Arp2/3, capping protein, actin, profilin, and ADF/cofilin, all involved in the shigella movements [193]. First, intracellular or extracellular signals activate the Rho-GTPase family that stimulates WASp/Scar protein. Then, WASp/Scar with Arp 2/3 and actin next to each other form a new branch [193]. Finally, the capping protein attaches to the distal side of the branch and terminates the new branch growth. In the Arp2/3 complex, a class of protein nucleation promoting factors (NPFs) facilitates actin activation. NPF leads to the activation of Arp2/3 which promotes the formation of the actin branch. One important WASP family of NFPs is N-WASP. By mimicking the activity of NPF, *Shigella* IcsA causes the release of autoinhibited N-WASP and activates Arp2/3, leading to actin assembly [194].

##### Cortactin

**Fig. 2:**
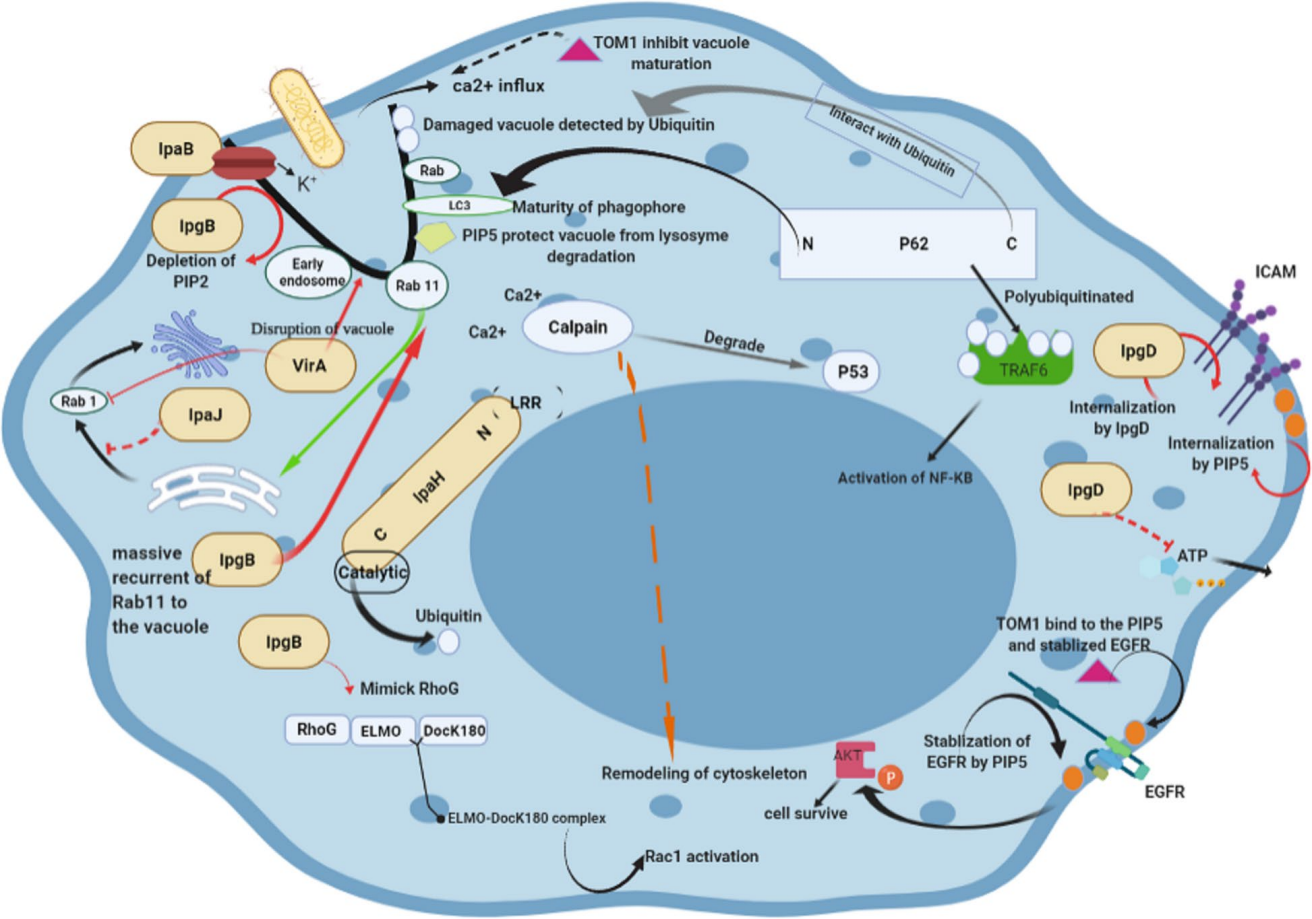
1 IpgB mediated to the block actin formation around vacuole. 2 IpaJ inhibit vesicle trafficking. 3 VirA lead to the disrupt of trafficking from RE to the Golgi and also mediated stabilized Rab1 in an inactivated form. 4 PIP5 regulate endosomal maturation and actin rearrangement and protect vacuole from lysosome degradation. 5 and 6 P62 can bind to the LC3 and also bind to the ubiquitin. 7 IpgB can mimic the role of RhoG and finally lead to the actin polymerization, massive recurrent of Rab11 to the vacuole. 8 PIP5 can activate Akt that mediated cell to survive. 9 TOM1 lead to lagging EGFR degradation. 10 IpgD can inhibit ATP releasing by cell as an inflammatory signal. 11 ICAM as a leukocyte receptor affected by IpgD and mediated to the internalization and degradation. 12 TOM1 inhibit vacuole maturation. IpaB, invasion plasmid antigen B; IpaJ, cysteine protease; IpgB, effector protein involved in *Shigella* invasion of host cells; IpaH, invasion plasmid antigen H *gene* sequence; Rab, Ras-related protein in brain; TOM 1, target of myb-1; RhoG, Ras homology growth related; ELMO, engulfment and cell motility protein; Dock180, dedicator of cytokinesis; ICAM, intercellular adhesion molecule; Akt, protein kinase B

After *the Shigella* invasion, the Src family is activated, and this leads to the recurrence and phosphorylation of cortactin. Cortactin is a substrate of the Src family that involves *the Shigella* entry process and regulates actin rearrangement. Cortactin has two domains: C-terminal and N-terminal. N-terminal acetylation (NTA) mediates the activation of Arp2/3 that is involved in the assembly of the actin branch. The next region after N-terminal is cortactin, which facilitates binding with the F-actin [195]. The SH3 domain of the C-terminal can bind with another activator of Arp2/3, which is called N-WASP. Therefore, cortactin may activate actin by either direct binding of NTA to the Arp2/3 or the recurrence of N-WASP by SH3 [196]. Cortactin may be recurred by the Rho GTPase family or microbial pathogens. Through glycine-rich IcsA, *Shigella* leads to the recurrence of N-WASP. The CRIB motif of N-WASP is enough to attach to the IcsA. In the next step, Cdc42 bounds to the CRIB motif, which regulates the ability of N-WASP to stimulate Arp2/3 and actin assembly [197, 198] (Fig. 2).

### Shiga toxin

**Fig. 3:**
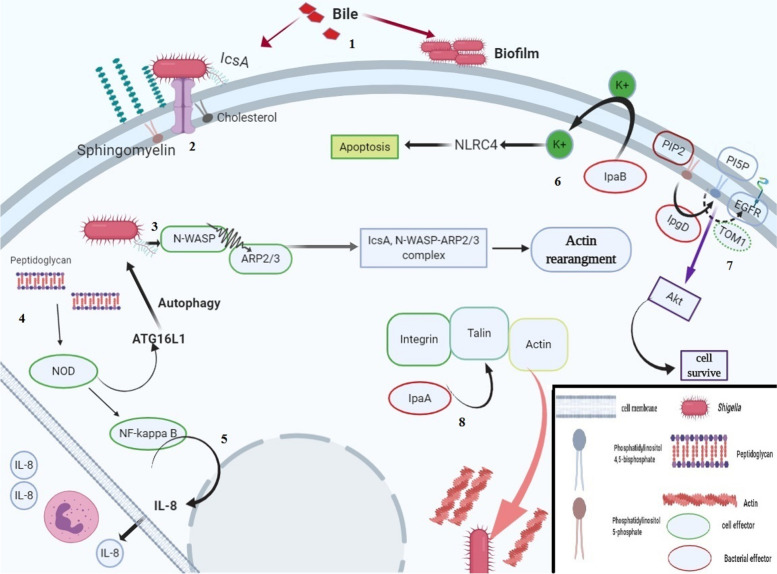
1 The presence of bile leads to the two important pathways, biofilm formation and IcsA expression. 2 Expression of IcsA leads to the attachment and internalization to the host cell. 3 Interaction of the IcsA with N-WASP finally leads to the actin rearrangement. 4 Interaction of NOD with *Shigella* peptidoglycan. 5 Sensing of peptidoglycan leads to activation of NF-kappa B that finally mediated transcription of IL-8. 6 IpaB can act as an ion channel. 7 IpgD mediated conversion of PIP2 to the PI5P that mediated activation of EGFR and summons of TOM1 to the lagging of EGFR degradation. 8 IpaA has a VBS that mediated binding to the talin and finally activated filopodial adhesin. IcsA, a 120-kDa outer membrane protein required for actin-based motility; NOD, nucleotide-binding oligomerization domain; PIP2, phosphatidylinositol 4,5-biphosphate; EGFR, epidermal growth factor receptor; N-WASP, neural Wiskott-Aldrich syndrome protein

Shiga toxin, a part of A-B toxin, is a pentamer B subunit that functions by connecting toxin to the host cell. Subunit A penetrates the host cell after connection with subunit B. Subunit B connects to the neutral glycolipid globotriaosylceramide (Gb3), and it is endocytosed. After endocytosis, it is transported to the Golgi and, conversely, is translocated to the ER. In the ER, subunit A enzymatically activates and transfers it to the cytosol to remove adenine from 28S RNA, which leads to inhibition of protein synthesis [199]. In the subunit A, the region between amino acids 248–251 mediates trypsin sensitivity and cleavages subunit A into A1 and A2 [200]. Gb3 as a significant receptor for toxin and cell line deficiency in Gb3 is insensitive to the toxin. In addition, Fabry’s disease with overexpressed Gb3 attenuates sensitivity to the Shiga toxin, perhaps due to the spread of toxin in the whole body instead of one position [201]. Shiga toxin also mediates Ca+ influx and ATP release from infected HeLa cells. ATP release mediates ATP signaling through purinergic receptor P2X, which in turn leads to Ca+ influx and cellular damage. Finally, it produces Shiga toxin containing microvesicle [202]. Shiga toxin mediates unfolded protein response mediated by ER and yields apoptosis for epithelial, lymphoid, and endothelial cells [203]. The toxin can be translocated and shed by stimulated blood cells. Translocation of toxin finally leads to its release by microvesicle and its attachment to a target cell such as a renal cell [204]. A human endothelial cell may have different amounts of Gb3 on the cell surface, and exposure to the LPS increases the amount of Gb3 sixfold. Interestingly, the level of Gb3 in renal cells is much more than the endothelial cell and may explain the sensitivity of the renal cell to the toxin [205] (Fig. 3).

### Immune response

In general, interference with the innate and adaptive immune responses in children during primary infection leads to the high susceptibility of children to shigellosis than adults [206]. The difference in human susceptibility to *Shigella* infection is rooted in the discrepancies between key components of the human innate defense barrier present in the colon [139, 206]. T3SS is the main weapon of *Shigella* to dampen host defenses. *Shigella* T3SS effectors target key cellular pathways of gut resident macrophages and enterocytes and modulate the important host cell functions [16]. *Shigella* T3SS effectors affect different signaling pathways involved in trafficking, cell viability, host cell actin cytoskeleton dynamics, and NF-κB-mediated inflammatory pathways [42]. On the other hand, *Shigella* leads to the reprogramming of gene expression in infected enterocytes and contributes to the downregulation of CCL20 production. CCL20 is a chemokine mediating DCs recruitment [207].

One mechanism for blocking the spread of cell-to-cell *Shigella* is GTP-binding protein (Septin). Septin has a role in cell division, but this protein can also activate through actin tail produced via cytoplasmic bacteria. TNF-α may stimulate the formation of septin and surround cytoplasmic bacteria that form the actin tail [208]. In other words, septins make a cage-like structure to contain the free movement of *Shigella*. Septin can sense micron-scale membrane curvature and bring the proteins back to the bacterial position. Cardiolipin with anionic properties has the presence on the bacterial division site. Cardiolipin via curvature property may trigger activation and migration of septin to the bacterial location [209]. Septin recruits to the IcsA position that mediates actin polymerization. Adaptor p62 protein also recurs to the septin location and mediates autophagy [210]. Cardiolipin is present in both the inner and outer membranes of the cell. PbgA as a transporter takes cardiolipin from the inner to outer cell membrane. The mutant of pbgA appears to be devoid of cardiolipin in the outer membrane cell. pgbA mutation cannot keep IcsA properly fixed in the outer membrane. Altogether, cardiolipin in the inner membrane is responsible for cell division and in the outer membrane responsible for properly localized IcsA [211]. IgG against IcsA and IpaB is shown to be protective, and it reduces the severity of *S. flexneri* infection [212]. Paneth cells secrete antimicrobial peptides such as LL-37 and defensin. Interestingly, *S. flexneri* can downregulate the secretion of this antimicrobial peptide. MxiE, as a bacterial regulator, can also regulate innate immune responses and suppress the expression of the antimicrobial peptide by intestinal cells. *S. flexneri* mediates suppression of chemokine CCL20 [139, 206].

Neutrophil destroys *Shigella* intracellularly or extracellularly; in the intracellular mode, it occurs following the engulfment of *Shigella* into vacuole present in the lysosome and is digested [213]. In the extracellular mode, *Shigella* is trapped and killed using neutrophil extracellular trap (NET). A critical component of NET is elastase that mediates the destruction of both outer membrane protein and virulence factor of *Shigella* in a lower concentration proportional to other bacterial proteins [214, 215]. After the interaction of LPS-TLR4, pentraxin 3 production is stimulated. Pentraxin 3 as a long pentraxin together with a short pentraxin such as C-reactive protein (CRP) forms acute-phase proteins. Pentraxin can bind with *Shigella* and interfere with *Shigella* epithelial invasion [216]. After the entrance of *S. flexneri* inside the macrophage, 34 KD outer membrane protein (OMP) can be detected by TLR-2. OMP induces expression of TRAF6 and MyD88 and facilitates the phosphorylation and activation of p38. It can also stimulate macrophages to produce chemokine and cytokine and upregulate the expression of MHC-II. All of these features are dependent on the expression of TLR-2 on the macrophage surface [217]. The 34 KD OMP exposed on the surface and antigenically conserved in *S. flexneri* mediates the induction of proinflammatory cytokines in macrophage, and it plays a protective role in stimulating an immune response [218]. In primary infection due to missing T-cell response, ultimately, eradication of *Shigella* failed; however, in the case of secondary infection, Th17 produces IL-17A which yields restricted bacterial infection.

**Fig. 4:**
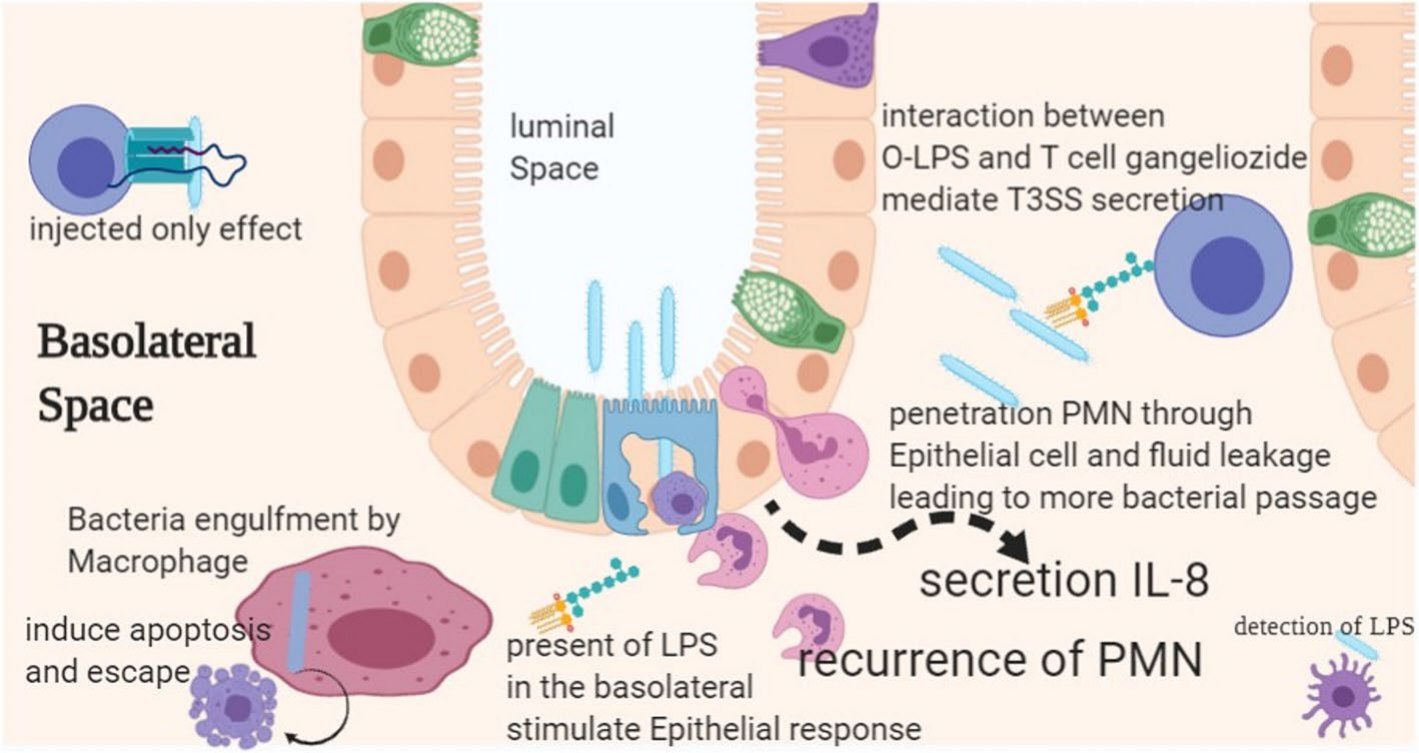
Penetration of the *Shigella* to the basolateral space and its consequence to invasion and immune stimulation

Interestingly, Th17 induction shows a stronger response over Th1 [219]. Although the adaptive immune response has a significant role in controlling intracellular infection, CD8+ cell as an adaptive immune cell has minor protection against *S. flexneri* infection. Interestingly, T-cell response can be attenuated when APC is infected with *S. flexneri* [220]. Although many vaccines have been proposed so far, only vaccines against O-polysaccharide were developed, and they ensure almost 2-year protection. This vaccine has functional capabilities and mediates serum bactericidal activities [221, 222]. However, this protectivity by IgG is serogroup specific against shigellosis. The presence of specific IgG and IgA leads to a protective effect against homologous *Shigella* species [222–224] (Fig. 4). *S. flexneri* can induce hyperinflammatory response through EGFR and NOD2. *Shigella* is inducing indoleamine 2,3-dioxygenases 1 (IDO1) by EGFR and NOD2. The above measure and the host cell response mediate immune hemostasis and disrupt IDO1 production through EGFR abrogation, and NOD2 signaling leads to imbalanced response as well as disrupted colon epithelial barrier and cytokine response [225]. Thus, *S. flexneri* induces IDO1 to mediate immune hemostasis, modulate cytokine secretion, and reduce cytokine consequences.

### Horizontal gene exchange in the human gut

Horizontal gene transfer (HGT) is the lateral exchange of genes between organisms and has been revealed for various organisms such as bacteria and viruses [226]. In total, the three main mechanisms of HGT are transformation, transduction, and conjugation. Conjugation is the most studied HGT mechanism in the human intestine, and it requires cell-to-cell contact [227]. Plasmids and the mobile genetic elements can be transferred through conjugative machinery. Bacteria in the gut environment have suitable conditions such as stable temperature, sufficient and permanent food resources, fixed physiological conditions, a large number of phages and bacterial cells, and plenty of opportunities for horizontal gene exchange [228]. It is revealed that the frequency of HGT in infants’ meconium and early fecal samples is higher than that in adults [229]. Genes that are widely transferred among bacterial genera and species encode proteins involved in fitness and multiple cycle-like alterations of gene expression. Prokaryotes of intestinal microbiome are reservoir of closely related antimicrobial resistance genes [228]. Antibiotic resistance genes spread between bacteria in gut environments through HGT and expression of resistance genes from other strains. Notably, HGTs along with the bacteriophages, conjugative transposons, plasmids, and integrons have the main role in the transfer of genes and acquisition of pathogenicity by pathogenic human enteric pathobionts, and it leads to the expansion of virulence traits and antibiotic resistance [228].

## Conclusion

*Shigella* is an essential agent in bacillary dysentery that has induced millions of diarrhea cases all around the world. *Shigella* can be transmitted through water and food, and it threatens children’s lives. After passing through the gastrointestinal, *Shigella* must be ready to face microbial flora. Since the microbial flora is adapted to the physiologic condition, *Shigella* should change this condition to reduce the compatibility of this flora. In the next step, changing circumstances by proteins effector ensures superiority to *Shigella*. This condition should not be continuous because of the mediated inflammation and the recurrent PMN; thus, it is the primary inflammation-mediated disorder in the mucous membrane that leads to the penetration of *Shigella* to the submucosal position. After passing to the submucosa, *Shigella* is engulfed by APC which stands firm against pathogens. However, this is not a problem for *Shigella* because it can modulate digestion.

Furthermore, *Shigella* can stimulate APC to induce apoptosis, but not before full duplication. Nevertheless, identifying the *Shigella* lifestyle and behavioral pattern leads to the recognition receptor, ligand, effector proteins, immune response modulators, and immune responses that mediate immunity. So far, several studies regarding immune response against *Shigella* mediates are done. One study demonstrated that infection with *Plesiomonas shigelloides* by identical LPS properties to the *Shigella sonnei* mediated protection against shigellosis [230]. Another strategy uses the outer membrane vesicle as a transporter of the *Shigella* antigen; this strategy is useful for other bacterial cells. Results demonstrated efficiency, similar to the live vaccine [231, 232]. Other studies use formalin as inactivated bacteria to induce immune responses. The result demonstrated the efficiency of the vaccine at the clinical trial phase 1 [233]. A new strategy to produce immunity is the subcellular vaccine, composed of LPS and plasmid that mediated invasion. This type of vaccine may produce proper protection against shigellosis [234]. One strategy that was formerly used and nowadays is reused by scholars is phage therapy. Phage therapy can be applied in a single- or multidose manner or monovalent or cocktail. Many studies have shown that the phage can specifically kill the pathogens [235–238]. Phage can detect and destroy *Shigella* and reduce shedding, without side effects on microbial flora. Phage can be useful as an antibiotic without side effects [239]. Altogether, so far, O-polysaccharide antigen has exhibited a better protective immune response and remains the candidate for the vaccine. Identification and mechanism of bacterial entry and pathogenicity play an essential role in vaccination against *Shigella*. So, in this review, our study explained how *Shigella* could enter the gastrointestinal and finally penetrate the submucosa, be engulfed by APC, escape APC, and evade the immune response. Altogether, O-polysaccharide is an essential factor in stimulating a protective immune response. For further research in the future, it is recommended that this factor be considered as having an influential role in full protection. In addition, a survey of the O-polysaccharide in the pathogenesis of *Shigella* is recommended.

## Data Availability

All data generated or analyzed during this study are included in this published article.

## Abbreviations

T3SS: *Type-three secretion system*
IFN-γ: *Interferon-gamma*
TNF-α: *Tumor necrosis factor-α*
MALT: Mucosal-associated lymphoid tissue
PMN: Polymorphonuclear neutrophils
DCs: Dendritic cells
NOD1: Nucleotide-binding oligomerization domain-1
RIP2: Receptor-interacting serine/threonine-protein kinase 2
TGF-β: Tumor growth factor-β
*NF-*κ*B*: Nuclear *factor* kappa-B
ICAM-1: Intracellular adhesion molecule-1
LPS: *Lipopolysaccharides*
ERK: Extracellular signal-regulated kinase
*PAMPs*: Pathogen-associated molecular patterns
PATR: Passenger-associated transport repeat
SPATE: Serine protease autotransporters of Enterobacteriaceae
TLR-2: Toll-like receptor 2
IAPs: Integrin-associated proteins
ATG: Autophagy-related proteins
TECPR1: Tectonic beta-propeller repeat-containing 1
PI5P: Phosphatidylinositol 5-phosphate
FNR: Fumarate and nitrate
GEF: Guanine nucleotide exchange factor activity
TRAF6: Tumor necrosis factor receptor-associated factor 6
GBP: Guanylate-binding protein

## References

[1] Tickell KD (2017). Identification and management of *Shigella* infection in children with diarrhoea: a systematic review and meta-analysis. Lancet Glob Health.

[2] Ashkenazi S (2004) *Shigella* infections in children: new insights. In: Seminars in pediatric infectious diseases. WBSaunders 5(12):246–252 10.1053/j.spid.2004.07.005 15494948

[3] Gharpure R (2021). Disparities in incidence and severity of *Shigella* Infections among children—Foodborne Diseases Active Surveillance Network (FoodNet), 2009-2018. J Pediatr Infect Dis Soc.

[4] Cheun H-I (2010). Infection status of hospitalized diarrheal patients with gastrointestinal protozoa, bacteria, and viruses in the Republic of Korea. Korean J Parasitol.

[5] Hawash YA, Ismail KA, Almehmadi M (2017). High frequency of enteric protozoan, viral, and bacterial potential pathogens in community-acquired acute diarrheal episodes: evidence based on results of luminex gastrointestinal pathogen panel assay. Korean J Parasitol.

[6] Kotloff KL (1999). Global burden of *Shigella* infections: implications for vaccine development and implementation of control strategies. Bull World Health Organ.

[7] Ud-Din A, Wahid S (2014). Relationship among *Shigella* spp. and enteroinvasive *Escherichia coli* (EIEC) and their differentiation. Braz. J Microbiol.

[8] Pormohammad A et al (2019) Prevalence of antibiotic resistance in *Escherichia coli* strains simultaneously isolated from humans, animals, food, and the environment: a systematic review and meta-analysis. Infect Drug Resist 12:1181–1197 10.2147/IDR.S201324 PMC6512575 31190907

[9] Zhu Z (2021). Virulence factors and molecular characteristics of *Shigella flexneri* isolated from calves with diarrhea. BMC Microbiol.

[10] Alemu A (2019). Prevalence, associated risk factors and antimicrobial susceptibility patterns of *Shigella* infections among diarrheic pediatric population attending at Gondar town healthcare institutions, northwest Ethiopia. Trop Dis Travel Med Vaccines.

[11] Khalil IA (2018). Morbidity and mortality due to *Shigella* and enterotoxigenic *Escherichia coli* diarrhoea: the Global Burden of Disease Study 1990–2016. Lancet Infect Dis.

[12] Das JK (2013). Antibiotics for the treatment of cholera, *Shigella* and *Cryptosporidium* in children. BMC Public Health.

[13] Fuchs A (2018). Reviewing the WHO guidelines for antibiotic use for sepsis in neonates and children. Paediatr Int Child Health.

[14] Kaminski RW, Oaks EV (2009). Inactivated and subunit vaccines to prevent shigellosis. Expert Rev Vaccines.

[15] Man AL, Prieto-Garcia ME, Nicoletti C (2004). Improving M cell mediated transport across mucosal barriers: do certain bacteria hold the keys?. Immunology.

[16] Ashida H, Mimuro H, Sasakawa C (2015). *Shigella* manipulates host immune responses by delivering effector proteins with specific roles. Front Immunol.

[17] Costa TR (2015). Secretion systems in gram-negative bacteria: structural and mechanistic insights. Nat Rev Microbiol.

[18] Delepelaire P (2004). Type I secretion in gram-negative bacteria. Biochimica et Biophysica Acta (BBA)-Molecular. Cell Res.

[19] Thomas S, Holland IB, Schmitt L (2014). The type 1 secretion pathway—the hemolysin system and beyond. Biochimica et Biophysica Acta (BBA)-Molecular. Cell Res.

[20] Green ER, Mecsas J (2016). Bacterial secretion systems: an overview. Microbiol Spect.

[21] Büttner D (2012). Protein export according to schedule: architecture, assembly, and regulation of type III secretion systems from plant-and animal-pathogenic bacteria. Microbiol Mol Biol Rev.

[22] Muthuramalingam M (2021). The *Shigella* type III secretion system: an overview from top to bottom. Microorganisms.

[23] Linden S (2008). Mucins in the mucosal barrier to infection. Mucosal Immunol.

[24] Smirnova MG, Birchall JP, Pearson JPJC (2000). TNF-alpha in the regulation of MUC5AC secretion: some aspects of cytokine-induced mucin hypersecretion on the in vitro model. Cytokine.

[25] Enss M-L (2000). Proinflammatory cytokines trigger MUC gene expression and mucin release in the intestinal cancer cell line LS180. Inflamm Res.

[26] Sperandio B (2013). Virulent *Shigella flexneri* affects secretion, expression, and glycosylation of gel-forming mucins in mucus-producing cells. Infect Immun.

[27] Corr SC (2008). M-cells: origin, morphology and role in mucosal immunity and microbial pathogenesis. FEMS Immunol Med Microbiol.

[28] Perdomo O (1994). Acute inflammation causes epithelial invasion and mucosal destruction in experimental shigellosis. J Exp Med.

[29] Ranganathan S (2019). Evaluating *Shigella flexneri* pathogenesis in the human enteroid model. Infect Immun.

[30] Zychlinsky A (1994). Interleukin 1 is released by murine macrophages during apoptosis induced by *Shigella flexneri*. J Clin Invest.

[31] Groeger S, Meyle J (2019). Oral mucosal epithelial cells. Front Immunol.

[32] Saxena M, Yeretssian G (2014). NOD-like receptors: master regulators of inflammation and cancer. Front Immunol.

[33] Phalipon A, Sansonetti PJ, c. biology (2007). Shigella’s ways of manipulating the host intestinal innate and adaptive immune system: a tool box for survival?. Immunol Cell Biol.

[34] Shin WG (2018). Infection of human intestinal epithelial cells by invasive bacteria activates NF-κB and increases ICAM-1 expression through NOD1. Korean J Intern Med.

[35] Girardin SE (2001). CARD4/Nod1 mediates NF-κB and JNK activation by invasive Shigella flexneri. EMBO Rep.

[36] Köhler H (2002). *Shigella flexneri* interactions with the basolateral membrane domain of polarized model intestinal epithelium: role of lipopolysaccharide in cell invasion and in activation of the mitogen-activated protein kinase ERK. Infect Immun.

[37] García-Weber D (2018). ADP-heptose is a newly identified pathogen-associated molecular pattern of Shigella flexneri. EMBO Rep.

[38] Gaudet RG (2017). Innate recognition of intracellular bacterial growth is driven by the TIFA-dependent cytosolic surveillance pathway. Cell Rep.

[39] Belotserkovsky I (2018). Glycan-glycan interaction determines *Shigella* tropism toward human T lymphocytes. MBio..

[40] Pinaud L (2017). Injection of T3SS effectors not resulting in invasion is the main targeting mechanism of Shigella toward human lymphocytes. Proc Natl Acad Sci.

[41] Foletta VC, Segal DH, Cohen DR (1998). Transcriptional regulation in the immune system: all roads lead to AP-1. J Leukoc Biol.

[42] Paciello I (2013). Intracellular Shigella remodels its LPS to dampen the innate immune recognition and evade inflammasome activation. Proc Natl Acad Sci.

[43] Zumsteg AB (2014). IcsA is a *Shigella flexneri* adhesin regulated by the type III secretion system and required for pathogenesis. Cell Host Microbe.

[44] Qin J (2020). The virulence domain of *Shigella* IcsA contains a subregion with specific host cell adhesion function. PLoS One.

[45] Brandon LD (2003). IcsA, a polarly localized autotransporter with an atypical signal peptide, uses the Sec apparatus for secretion, although the Sec apparatus is circumferentially distributed. Mol Microbiol.

[46] Scribano D et al (2014) Polar localization of PhoN2, a periplasmic virulence-associated factor of *Shigella flexneri*, is required for proper IcsA exposition at the old bacterial pole. PLoS One 9(2):e90230 10.1371/journal.pone.0090230 PMC3937361 24587292

[47] Pope LM (1995). Increased protein secretion and adherence to HeLa cells by *Shigella* spp. following growth in the presence of bile salts. Infect Immun.

[48] Faherty CS (2012). *Shigella flexneri* effectors OspE1 and OspE2 mediate induced adherence to the colonic epithelium following bile salts exposure. Mol Microbiol.

[49] Kim M (2009). Bacteria hijack integrin-linked kinase to stabilize focal adhesions and block cell detachment. Nature..

[50] Nickerson KP (2017). Analysis of *Shigella flexneri* resistance, biofilm formation, and transcriptional profile in response to bile salts. Infect Immun.

[51] Sharahi JY (2019). Advanced strategies for combating bacterial biofilms. J Cell Physiol.

[52] Egile C (1999). Activation of the CDC42 effector N-WASP by the *Shigella flexneri* IcsA protein promotes actin nucleation by Arp2/3 complex and bacterial actin-based motility. J Cell Biol.

[53] Fawcett J, Pawson TJS (2000). N-WASP tegulation--the sting in the tail. Science..

[54] Carlier M-F (1999). Signalling to actin: the Cdc42-N-WASP-Arp2/3 connection. Chem Biol.

[55] Suzuki T (2002). Neural Wiskott–Aldrich syndrome protein (N-WASP) is the specific ligand for *Shigella* VirG among the WASP family and determines the host cell type allowing actin-based spreading. Cell Microbiol.

[56] Suzuki T, Saga S, Sasakawa C (1996). Functional analysis of *Shigella* VirG domains essential for interaction with vinculin and actin-based motility. J Biol Chem.

[57] Henderson IR (1999). Characterization of Pic, a secreted protease of *Shigella flexneri* and Enteroaggregative *Escherichia coli*. Infect Immun.

[58] Dautin NJT (2010). Serine protease autotransporters of enterobacteriaceae (SPATEs): biogenesis and function. Toxins..

[59] Ruiz-Perez F (2011). Serine protease autotransporters from *Shigella flexneri* and pathogenic *Escherichia coli* target a broad range of leukocyte glycoproteins. Proc Natl Acad Sci.

[60] Gutierrez-Jimenez J, Arciniega I, Navarro-García F (2008). The serine protease motif of Pic mediates a dose-dependent mucolytic activity after binding to sugar constituents of the mucin substrate. Microb Pathog.

[61] Dutta PR (2002). Functional comparison of serine protease autotransporters of Enterobacteriaceae. Infect Immun.

[62] Harrington SM (2009). The Pic protease of enteroaggregative *Escherichia coli* promotes intestinal colonization and growth in the presence of mucin. Infect Immun.

[63] Veenendaal AK (2007). The type III secretion system needle tip complex mediates host cell sensing and translocon insertion. Mol Microbiol.

[64] Lafont F (2002). Initial steps of Shigella infection depend on the cholesterol/sphingolipid raft-mediated CD44–IpaB interaction. EMBO J.

[65] Epler CR (2012). Ultrastructural analysis of IpaD at the tip of the nascent MxiH type III secretion apparatus of *Shigella flexneri*. J Mol Biol.

[66] Dickenson NE (2011). Conformational changes in IpaD from *Shigella flexneri* upon binding bile salts provide insight into the second step of type III secretion. Biochemistry..

[67] Martinez-Argudo I, Blocker AJ (2010). The *Shigella* T3SS needle transmits a signal for MxiC release, which controls secretion of effectors. Mol Microbiol.

[68] Epler CR (2009). Liposomes recruit IpaC to the *Shigella flexneri* type III secretion apparatus needle as a final step in secretion induction. Infect Immun.

[69] Yang Y (2019). Recent advances in the mechanisms of NLRP3 inflammasome activation and its inhibitors. Cell Death Dis.

[70] Senerovic L (2012). Spontaneous formation of IpaB ion channels in host cell membranes reveals how *Shigella* induces pyroptosis in macrophages. Cell Death Dis.

[71] Skoudy A (2000). CD44 binds to the *Shigella* IpaB protein and participates in bacterial invasion of epithelial cells. Cell Microbiol.

[72] Mounier J (2009). The IpaC carboxyterminal effector domain mediates Src-dependent actin polymerization during *Shigella* invasion of epithelial cells. PLoS Pathog.

[73] Frame MC (2002). Src in cancer: deregulation and consequences for cell behaviour. Biochimica et Biophysica Acta (BBA)-Reviews on. Cancer..

[74] Lunelli M (2009). IpaB–IpgC interaction defines binding motif for type III secretion translocator. Proc Natl Acad Sci.

[75] Lokareddy RK (2010). Combination of two separate binding domains defines stoichiometry between type III secretion system chaperone IpgC and translocator protein IpaB. J Biol Chem.

[76] Carayol N, Van Nhieu GT (2013). Tips and tricks about *Shigella* invasion of epithelial cells. Curr Opin Microbiol.

[77] Nothelfer K (2014). B lymphocytes undergo TLR2-dependent apoptosis upon *Shigella* infection. J Exp Med.

[78] Klapholz B, Brown NH (2017). Talin–the master of integrin adhesions. J Cell Sci.

[79] Calderwood DA, Campbell ID, Critchley DR (2013). Talins and kindlins: partners in integrin-mediated adhesion. Nat Rev Mol Cell Biol.

[80] Liu J (2015). Talin determines the nanoscale architecture of focal adhesions. Proc Natl Acad Sci.

[81] Valencia-Gallardo C (2019). *Shigella* IpaA binding to talin stimulates filopodial capture and cell adhesion. Cell Rep.

[82] Izard T, Tran Van Nhieu G, Bois PRJ (2006). *Shigella* applies molecular mimicry to subvert vinculin and invade host cells. J Cell Biol.

[83] Feng Y (2014). The machinery of macroautophagy. Cell Res.

[84] Travassos LH (2010). Nod1 and Nod2 direct autophagy by recruiting ATG16L1 to the plasma membrane at the site of bacterial entry. Nat Immunol.

[85] Huang J, Brumell JHJNRM (2014). Bacteria–autophagy interplay: a battle for survival. Nat Rev Microbiol.

[86] Ogawa M (2003). IcsB, secreted via the type III secretion system, is chaperoned by IpgA and required at the post-invasion stage of *Shigella* pathogenicity. Mol Microbiol.

[87] Baxt LA, Goldberg MB (2014). Host and bacterial proteins that repress recruitment of LC3 to *Shigella* early during infection. PLoS One.

[88] Ho H-YH (2004). Toca-1 mediates Cdc42-dependent actin nucleation by activating the N-WASP-WIP complex. Cell..

[89] Ogawa M (2011). A Tecpr1-dependent selective autophagy pathway targets bacterial pathogens. Cell Host Microbe.

[90] Campbell-Valois F-X (2015). Escape of actively secreting *Shigella flexneri* from ATG8/LC3-positive vacuoles formed during cell-to-cell spread is facilitated by IcsB and VirA. MBio..

[91] Niebuhr K (2002). Conversion of PtdIns (4, 5) P2 into PtdIns (5) P by the *S. flexneri* effector IpgD reorganizes host cell morphology. EMBO J.

[92] Azimi T (2020). Molecular mechanisms of *Salmonella* effector proteins: a comprehensive review. Infect Drug Resist.

[93] Ramel D (2011). *Shigella flexneri* infection generates the lipid PI5P to alter endocytosis and prevent termination of EGFR signaling. Sci Signal.

[94] Boal F (2015). TOM1 is a PI5P effector involved in the regulation of endosomal maturation. J Cell Sci.

[95] Pendaries C (2006). PtdIns (5) P activates the host cell PI3-kinase/Akt pathway during *Shigella flexneri* infection. EMBO J.

[96] Janmey PA, Lindberg UJNRMCB (2004). Cytoskeletal regulation: rich in lipids. Nat Rev Mol Cell Biol.

[97] Nasser A (2019). *Staphylococcus aureus* versus neutrophil: scrutiny of ancient combat. Microb Pathog.

[98] Grant BD, Donaldson JG (2009). Pathways and mechanisms of endocytic recycling. Nat Rev Mol Cell Biol.

[99] Mellouk N (2014). *Shigella* subverts the host recycling compartment to rupture its vacuole. Cell Host Microbe.

[100] Konradt C (2011). The *Shigella flexneri* type three secretion system effector IpgD inhibits T cell migration by manipulating host phosphoinositide metabolism. Cell Host Microbe.

[101] Boal F (2016). PI5P triggers ICAM-1 degradation in *Shigella* infected cells, thus dampening immune cell recruitment. Cell Rep.

[102] Puhar A (2013). A *Shigella* effector dampens inflammation by regulating epithelial release of danger signal ATP through production of the lipid mediator PtdIns5P. Immunity..

[103] Van Nhieu GT (2003). Connexin-dependent inter-cellular communication increases invasion and dissemination of Shigella in epithelial cells. Nat Cell Biol.

[104] Dong N (2012). Structurally distinct bacterial TBC-like GAPs link Arf GTPase to Rab1 inactivation to counteract host defenses. Cell..

[105] Maurelli AT (1984). Temperature-dependent expression of virulence genes in *Shigella* species. Infect Immun.

[106] Van Nhieu GT (2013). Actin-based confinement of calcium responses during *Shigella* invasion. Nat Commun.

[107] Calle Y (2006). Inhibition of calpain stabilises podosomes and impairs dendritic cell motility. J Cell Sci.

[108] Romero S (2011). ATP-mediated Erk1/2 activation stimulates bacterial capture by filopodia, which precedes *Shigella* invasion of epithelial cells. Cell Host Microbe.

[109] Bergounioux J (2012). Calpain activation by the *Shigella flexneri* effector VirA regulates key steps in the formation and life of the bacterium's epithelial niche. Cell Host Microbe.

[110] Bonnet M, Van Nhieu GT, i. microbiology (2016). How *Shigella* utilizes Ca2+ jagged edge signals during invasion of epithelial cells. Front Cell Infect Microbiol.

[111] Sukumaran SK (2010). A soluble form of the pilus protein FimA targets the VDAC-hexokinase complex at mitochondria to suppress host cell apoptosis. Mol Cell.

[112] Marteyn B (2010). Modulation of *Shigella* virulence in response to available oxygen in vivo. Nature..

[113] Tinevez J-Y (2019). *Shigella*-mediated oxygen depletion is essential for intestinal mucosa colonization. Nat Microbiol.

[114] Huang Z (2009). Structural insights into host GTPase isoform selection by a family of bacterial GEF mimics. Nat Struct Mol Biol.

[115] Parsons JT, Horwitz AR, Schwartz MA (2010). Cell adhesion: integrating cytoskeletal dynamics and cellular tension. Nat Rev Mol Cell Biol.

[116] Handa Y (2007). *Shigella* IpgB1 promotes bacterial entry through the ELMO–Dock180 machinery. Nat Cell Biol.

[117] Brugnera E (2002). Unconventional Rac-GEF activity is mediated through the Dock180–ELMO complex. Nat Cell Biol.

[118] Bulgin R (2010). Bacterial guanine nucleotide exchange factors SopE-like and WxxxE effectors. Infect Immun.

[119] Alto NM (2006). Identification of a bacterial type III effector family with G protein mimicry functions. Cell.

[120] Zheng YJTibs (2001). Dbl family guanine nucleotide exchange factors. Trends Biochem Sci.

[121] Fukazawa A (2008). GEF-H1 mediated control of NOD1 dependent NF-κB activation by *Shigella* effectors. PLoS Pathog.

[122] Wortham BW (2007). Polyamines in bacteria: pleiotropic effects yet specific mechanisms. Adv Exp Med Biol.

[123] Gevrekci AÖJWJoM, Biotechnology (2017). The roles of polyamines in microorganisms. World J Microbiol Biotechnol.

[124] Jeong J-W (2018). Spermidine protects against oxidative stress in inflammation models using macrophages and zebrafish. Biomol Therapeut.

[125] Barbagallo M (2011). A new piece of the *Shigella* pathogenicity puzzle: spermidine accumulation by silencing of the speG gene [corrected]. PLoS One.

[126] Kayagaki N (2013). Noncanonical inflammasome activation by intracellular LPS independent of TLR4. Science.

[127] Knodler LA (2014). Noncanonical inflammasome activation of caspase-4/caspase-11 mediates epithelial defenses against enteric bacterial pathogens. Cell Host Microbe.

[128] Bergsbaken T, Fink SL, Cookson BTJNRM (2009). Pyroptosis: host cell death and inflammation. Nat Rev Microbiol.

[129] Hagar JA (2013). Cytoplasmic LPS activates caspase-11: implications in TLR4-independent endotoxic shock. Science.

[130] Ashida H, Kim M, Sasakawa CJCm (2014). Manipulation of the host cell death pathway by *Shigella*. Cell Microbiol.

[131] Carneiro LA (2009). *Shigella* induces mitochondrial dysfunction and cell death in nonmyleoid cells. Cell Host Microbe.

[132] Carneiro L (2008). Nod-like proteins in inflammation and disease. J Pathol: J Pathol Soc Great Britain Ireland.

[133] Kufer TA (2008). The pattern-recognition molecule Nod1 is localized at the plasma membrane at sites of bacterial interaction. Cell Microbiol.

[134] Tanabe T (2004). Regulatory regions and critical residues of NOD2 involved in muramyl dipeptide recognition. EMBO J.

[135] Hu Z (2013). Crystal structure of NLRC4 reveals its autoinhibition mechanism. Science.

[136] Miao EA (2010). Innate immune detection of the type III secretion apparatus through the NLRC4 inflammasome. Proc Natl Acad Sci.

[137] Rayamajhi M (2013). Cutting edge: mouse NAIP1 detects the type III secretion system needle protein. J Immunol.

[138] Yang J (2013). Human NAIP and mouse NAIP1 recognize bacterial type III secretion needle protein for inflammasome activation. Proc Natl Acad Sci.

[139] Sperandio B (2008). Virulent *Shigella flexneri* subverts the host innate immune response through manipulation of antimicrobial peptide gene expression. J Exp Med.

[140] Willingham SB (2007). Microbial pathogen-induced necrotic cell death mediated by the inflammasome components CIAS1/cryopyrin/NLRP3 and ASC. Cell Host Microbe.

[141] Suzuki T (2005). A novel caspase-1/toll-like receptor 4-independent pathway of cell death induced by cytosolic *Shigella* in infected macrophages. J Biol Chem.

[142] Kofoed EM, Vance REJN (2011). Innate immune recognition of bacterial ligands by NAIPs determines inflammasome specificity. Nature.

[143] Zhao Y (2016). Genetic functions of the NAIP family of inflammasome receptors for bacterial ligands in mice. J Exp Med.

[144] Campbell-Valois F-X (2014). A fluorescent reporter reveals on/off regulation of the *Shigella* type III secretion apparatus during entry and cell-to-cell spread. Cell Host Microbe.

[145] Ashida H (2007). *Shigella* chromosomal IpaH proteins are secreted via the type III secretion system and act as effectors. Mol Microbiol.

[146] Bell JK (2003). Leucine-rich repeats and pathogen recognition in Toll-like receptors. Trends Immunol.

[147] Norkowski S (2018). Bacterial LPX motif-harboring virulence factors constitute a species-spanning family of cell-penetrating effectors. Cell Mol Life Sci.

[148] Ashida H, Kim M, Sasakawa CJNRM (2014). Exploitation of the host ubiquitin system by human bacterial pathogens. Nat Rev Microbiol.

[149] Dupont N (2009). *Shigella* phagocytic vacuolar membrane remnants participate in the cellular response to pathogen invasion and are regulated by autophagy. Cell Host Microbe.

[150] Pankiv S (2007). p62/SQSTM1 binds directly to Atg8/LC3 to facilitate degradation of ubiquitinated protein aggregates by autophagy. J Biol Chem.

[151] Wooten MW (2005). The p62 scaffold regulates nerve growth factor-induced NF-κB activation by influencing TRAF6 polyubiquitination. J Biol Chem.

[152] Vallabhapurapu S, Karin MJAroi (2009). Regulation and function of NF-κB transcription factors in the immune system. Annu Rev Immunol.

[153] Hayden MS, Ghosh SJC (2008). Shared principles in NF-κB signaling. Cell.

[154] Fujita H (2014). Mechanism underlying IκB kinase activation mediated by the linear ubiquitin chain assembly complex. Mol Cell Biol.

[155] Lamothe B (2007). Site-specific Lys-63-linked tumor necrosis factor receptor-associated factor 6 auto-ubiquitination is a critical determinant of IκB kinase activation. J Biol Chem.

[156] Miyamoto SJCr (2011). Nuclear initiated NF-κB signaling: NEMO and ATM take center stage. Cell Res.

[157] Lopez-Montero N, Enninga JJCh (2017). *Shigella* stays on the move. Cell Host Microbe.

[158] Ashida H (2010). A bacterial E3 ubiquitin ligase IpaH9. 8 targets NEMO/IKKγ to dampen the host NF-κB-mediated inflammatory response. Nat Cell Biol.

[159] He Y, Hara H, Núñez GJTibs (2016). Mechanism and regulation of NLRP3 inflammasome activation. Trends Biochem Sci.

[160] Duda DM (2012). Structure of a glomulin-RBX1-CUL1 complex: inhibition of a RING E3 ligase through masking of its E2-binding surface. Mol Cell.

[161] Suzuki S (2014). Shigella IpaH7. 8 E3 ubiquitin ligase targets glomulin and activates inflammasomes to demolish macrophages. Proc Natl Acad Sci.

[162] De Jong MF (2016). *Shigella flexneri* suppresses NF-κB activation by inhibiting linear ubiquitin chain ligation. Nat Microbiol.

[163] Suzuki S (2018). *Shigella* hijacks the glomulin–cIAPs–inflammasome axis to promote inflammation. EMBO Rep.

[164] Zheng Z (2016). Bacterial E3 ubiquitin ligase IpaH4. 5 of *Shigella flexneri* targets TBK1 to dampen the host antibacterial response. J Immunol.

[165] Otsubo R (2019). *Shigella* effector IpaH 4.5 targets 19 S regulatory particle subunit RPN13 in the 26 S proteasome to dampen cytotoxic T lymphocyte activation. Cell Microbiol.

[166] D'Souza-Schorey C, Chavrier PJNrMcb (2006). ARF proteins: roles in membrane traffic and beyond. Nat Rev Mol Cell Biol.

[167] Burnaevskiy N (2015). Myristoylome profiling reveals a concerted mechanism of ARF GTPase deacylation by the bacterial protease IpaJ. Mol Cell.

[168] Stearns T (1990). ADP-ribosylation factor is functionally and physically associated with the Golgi complex. Proc Natl Acad Sci.

[169] Dobbs N (2015). STING activation by translocation from the ER is associated with infection and autoinflammatory disease. Cell Host Microbe.

[170] Six DA, Dennis EAJBeBA-M (2000). The expanding superfamily of phospholipase A2 enzymes: classification and characterization. Biochim Biophys Acta (BBA)-Mol Cell Biol Lipids.

[171] Linkous A, Yazlovitskaya EJCm (2010). Cytosolic phospholipase A2 as a mediator of disease pathogenesis. Cell Microbiol.

[172] Lu R et al (2015) *Shigella* effector OspB activates mTORC1 in a manner that depends on IQGAP1 and promotes cell proliferation. PLoS Pathog 11(10):e1005200 10.1371/journal.ppat.1005200 PMC4608727 26473364

[173] Mendoza MC, Er EE, Blenis JJTibs (2011). The Ras-ERK and PI3K-mTOR pathways: cross-talk and compensation. Trends Biochem Sci.

[174] Zurawski DV (2006). OspF and OspC1 are *Shigella flexneri* type III secretion system effectors that are required for postinvasion aspects of virulence. Infect Immun.

[175] Singer M, Sansonetti PJJTJoi (2004). IL-8 is a key chemokine regulating neutrophil recruitment in a new mouse model of *Shigella*-induced colitis. J Immunol.

[176] Kobayashi T (2013). The Shigella OspC3 effector inhibits caspase-4, antagonizes inflammatory cell death, and promotes epithelial infection. Cell Host Microbe.

[177] Harouz H (2015). Targeting of chromatin readers: a novel strategy used by the *Shigella flexneri* virulence effector OspF to reprogram transcription. Microbial Cell.

[178] Harouz H (2014). *Shigella flexneri* targets the HP1γ subcode through the phosphothreonine lyase OspF. EMBO J.

[179] Arbibe L (2007). An injected bacterial effector targets chromatin access for transcription factor NF-κB to alter transcription of host genes involved in immune responses. Nat Immunol.

[180] Zurawski DV (2009). *Shigella flexneri* type III secretion system effectors OspB and OspF target the nucleus to downregulate the host inflammatory response via interactions with retinoblastoma protein. Mol Microbiol.

[181] Jo K (2017). Host cell nuclear localization of *Shigella flexneri* effector OspF is facilitated by SUMOylation. J Microbiol Biotechnol.

[182] Goldfarb DS (2004). Importin α: a multipurpose nuclear-transport receptor. Trends Cell Biol.

[183] Zhao H (2019). The *Shigella* type three secretion system effector OspF invades host nucleus by binding host importin α1. World J Microbiol Biotechnol.

[184] Li Q, Verma IMJNRI (2002). NF-κB regulation in the immune system. Nat Rev Immunol.

[185] Kim DW (2005). The *Shigella flexneri* effector OspG interferes with innate immune responses by targeting ubiquitin-conjugating enzymes. Proc Natl Acad Sci.

[186] Sanada T (2012). The *Shigella flexneri* effector OspI deamidates UBC13 to dampen the inflammatory response. Nature.

[187] Nishide A (2013). Structural basis for the recognition of Ubc13 by the *Shigella flexneri* effector OspI. J Mol Biol.

[188] Mohanty P (2019). Deamidation disrupts native and transient contacts to weaken the interaction between UBC13 and RING-finger E3 ligases. ELife.

[189] Newton HJ et al (2010) The type III effectors NleE and NleB from enteropathogenic *E. coli* and OspZ from Shigella block nuclear translocation of NF-κB p65. PLoS Pathog 6(5):e1000898 10.1371/journal.ppat.1000898 PMC2869321 20485572

[190] Yao Q et al (2014) Structure and specificity of the bacterial cysteine methyltransferase effector NleE suggests a novel substrate in human DNA repair pathway. PLoS Pathog 10(11):e1004522 10.1371/journal.ppat.1004522 PMC4239114 25412445

[191] Zhang L (2012). Cysteine methylation disrupts ubiquitin-chain sensing in NF-κB activation. Nature.

[192] Zhang Y (2016). Identification of a distinct substrate-binding domain in the bacterial cysteine methyltransferase effectors NleE and OspZ. J Biol Chem.

[193] Pollard TD, Borisy GGJC (2003). Cellular motility driven by assembly and disassembly of actin filaments. Cell.

[194] Stradal TE, Schelhaas MJFl (2018). Actin dynamics in host–pathogen interaction. FEBS Lett.

[195] Daly RJJBJ (2004). Cortactin signalling and dynamic actin networks. Biochem J.

[196] Selbach M, Backert SJTim (2005). Cortactin: an Achilles’ heel of the actin cytoskeleton targeted by pathogens. Trends Microbiol.

[197] Rohatgi R, Ho H-yH, Kirschner MWJTJocb (2000). Mechanism of N-WASP activation by CDC42 and phosphatidylinositol 4, 5-bisphosphate. J Cell Biol.

[198] Miki H (1998). Induction of filopodium formation by a WASP-related actin-depolymerizing protein N-WASP. Nature.

[199] Sandvig KJTcsobpt (2005). The Shiga toxins: properties and action on cells. The comprehensive sourcebook of bacterial protein toxins.

[200] Melton-Celsa ARJMs, (2014). Shiga toxin (Stx) classification, structure, and function. Microbiol Spect.

[201] Cilmi SA (2006). Fabry disease in mice protects against lethal disease caused by Shiga toxin–expressing enterohemorrhagic *Escherichia coli*. J Infect Dis.

[202] Johansson KE (2019). Shiga toxin signals via ATP and its effect is blocked by purinergic receptor antagonism. Sci Rep.

[203] Tesh VL (2012). The induction of apoptosis by Shiga toxins and ricin. Curr Top Microbiol Immunol.

[204] Villysson A (2018). Shiga toxin interactions with microvesicles. J Extracell Vesicles.

[205] Obrig TG (1993). Endothelial heterogeneity in Shiga toxin receptors and responses. J Biol Chem.

[206] Brunner K (2019). *Shigella*-mediated immunosuppression in the human gut: subversion extends from innate to adaptive immune responses. Hum Vaccin Immunother.

[207] Pédron T, Thibault C, Sansonetti PJ (2003). The invasive phenotype of *Shigella flexneri* directs a distinct gene expression pattern in the human intestinal epithelial cell line Caco-2. J Biol Chem.

[208] Mostowy S (2010). Entrapment of intracytosolic bacteria by septin cage-like structures. Cell Host Microbe.

[209] Krokowski S (2018). Septins recognize and entrap dividing bacterial cells for delivery to lysosomes. Cell Host Microbe.

[210] Mostowy S (2011). p62 and NDP52 proteins target intracytosolic *Shigella* and *Listeria* to different autophagy pathways. J Biol Chem.

[211] Rossi RM (2017). Cardiolipin synthesis and outer membrane localization are required for *Shigella flexneri* virulence. MBio.

[212] McArthur MA, Maciel M, Pasetti MFJV (2017). Human immune responses against *Shigella* and enterotoxigenic *E. coli*: current advances and the path forward. Vaccine.

[213] Brinkmann V (2004). Neutrophil extracellular traps kill bacteria. science.

[214] Weinrauch Y (2002). Neutrophil elastase targets virulence factors of enterobacteria. Nature.

[215] Averhoff P (2008). Single residue determines the specificity of neutrophil elastase for *Shigella* virulence factors. J Mol Biol.

[216] Ciancarella V (2018). Role of a fluid-phase PRR in fighting an intracellular pathogen: PTX3 in *Shigella* infection. PLoS Pathog.

[217] Pore D (2010). 34 kDa MOMP of *Shigella flexneri* promotes TLR2 mediated macrophage activation with the engagement of NF-κB and p38 MAP kinase signaling. Mol Immunol.

[218] Pore D (2009). Purification and characterization of an immunogenic outer membrane protein of *Shigella flexneri* 2a. Vaccine.

[219] Sellge G (2010). Th17 cells are the dominant T cell subtype primed by *Shigella flexneri* mediating protective immunity. J Immunol.

[220] Jehl SP (2011). Antigen-specific CD8+ T cells fail to respond to *Shigella flexneri*. Infect Immun.

[221] Cohen D (2019). Serum IgG antibodies to Shigella lipopolysaccharide antigens–a correlate of protection against shigellosis. Hum Vaccin Immunother.

[222] Cohen D, Muhsen KJHv (2019). Vaccines for enteric diseases. Hum Vaccin Immunother.

[223] Cohen D (1991). Prospective study of the association between serum antibodies to lipopolysaccharide O antigen and the attack rate of shigellosis. J Clin Microbiol.

[224] Mani S (2019). Role of antigen specific T and B cells in systemic and mucosal immune responses in ETEC and *Shigella* infections, and their potential to serve as correlates of protection in vaccine development. Vaccine.

[225] Mukherjee T (2019). Epidermal growth factor receptor–responsive indoleamine 2, 3-dioxygenase confers immune homeostasis during *Shigella flexneri* infection. J Infect Dis.

[226] Huddleston JR (2014). Horizontal gene transfer in the human gastrointestinal tract: potential spread of antibiotic resistance genes. Infect Drug Resist.

[227] Lerner A, Matthias T, Aminov R (2017). Potential effects of horizontal gene exchange in the human gut. Front Immunol.

[228] Boto L, Pineda M, Pineda R (2019). Potential impacts of horizontal gene transfer on human health and physiology and how anthropogenic activity can affect it. FEBS J.

[229] Gosalbes M (2016). High frequencies of antibiotic resistance genes in infants’ meconium and early fecal samples. Journal of developmental origins of health and disease. J Dev Orig Health Dis.

[230] Sack DA (1994). Is protection against shigellosis induced by natural infection with Plesiomonas shigelloides?. Lancet.

[231] Nasser A, Zamirnasta M, Jalilian FAJBbra (2014). Bacterial nanoparticle as a vaccine for meningococcal disease. Biosci Biotechnol Res Asia.

[232] Fries LF (2001). Safety and immunogenicity of a proteosome-*Shigella flexneri* 2a lipopolysaccharide vaccine administered intranasally to healthy adults. Infect Immun.

[233] McKenzie R (2006). Safety and immunogenicity of an oral, inactivated, whole-cell vaccine for *Shigella sonnei*: preclinical studies and a phase I trial. Vaccine.

[234] Oaks EV, Turbyfill KRJV (2006). Development and evaluation of a *Shigella flexneri* 2a and *S. sonnei* bivalent invasin complex (Invaplex) vaccine. Vaccine.

[235] Nasser A (2019). Specification of bacteriophage isolated against clinical methicillin-resistant *staphylococcus aureus*. Osong Public Health Res Perspect.

[236] Azizian R (2015). Sewage as a rich source of phage study against *Pseudomonas aeruginosa* PAO. Biologicals.

[237] Rezaei F (2014). Using phage as a highly specific antibiotic alternative against methicillin resistance *Staphylococcus aureus* (MRSA). Biosci Biotechnol Res Asia.

[238] Azimi T (2019). Phage therapy as a renewed therapeutic approach to mycobacterial infections: a comprehensive review. Infect Drug Resist.

[239] Mai V (2015). Bacteriophage administration significantly reduces *Shigella* colonization and shedding by *Shigella*-challenged mice without deleterious side effects and distortions in the gut microbiota. Bacteriophage.

